# Differential Expression of the β3 Subunit of Voltage-Gated Ca^2+^ Channel in Mesial Temporal Lobe Epilepsy

**DOI:** 10.1007/s12035-023-03426-4

**Published:** 2023-06-21

**Authors:** Christina Kjær, Oana Palasca, Guido Barzaghi, Lasse K. Bak, Rúna K. J. Durhuus, Emil Jakobsen, Louise Pedersen, Emil D. Bartels, David P. D. Woldbye, Lars H. Pinborg, Lars Juhl Jensen

**Affiliations:** 1 Biomedical Laboratory Science, Department of Technology, Faculty of Health and Technology, University College Copenhagen, Sigurdsgade 26, 1St, 2200 Copenhagen, Denmark; 2 Department of Drug Design and Pharmacology, Faculty of Health and Medical Sciences, University of Copenhagen, 2100 Copenhagen, Denmark; 3 Disease Systems Biology Program, Faculty of Health and Medical Sciences, Novo Nordisk Foundation Center for Protein Research, University of Copenhagen, Copenhagen, Denmark; 4 Dept. of Clinical Biochemistry, 2600 RigshospitaletCopenhagen, Denmark; 5 Department of Neuroscience, University of Copenhagen, 2200 Copenhagen, Denmark; 6 Epilepsy Clinic & Neurobiology Research Unit, Copenhagen University Hospital, University of Copenhagen, 2100 Copenhagen, Denmark; 7 Department of Clinical Medicine, University of Copenhagen, Copenhagen, Denmark; 8 European Molecular Biology Laboratory (EMBL), Genome Biology Unit, Heidelberg, Germany; 9 Faculty of Biosciences, Collaboration for Joint PhD Degree Between EMBL and Heidelberg University, Heidelberg, Germany; 10 Specific Pharma A/S, Borgmester Christiansens Gade 40, 2450 Copenhagen, SV Denmark; 11 Takeda Pharma A/S, Delta Park 45, 2665 Vallensbaek Strand, Denmark

**Keywords:** Mesial temporal lobe epilepsy, mTLE, Transcriptome analysis, Unbiased drug target identification, Target validation, CACNB3

## Abstract

The purpose of this study was to identify and validate new putative lead drug targets in drug-resistant mesial temporal lobe epilepsy (mTLE) starting from differentially expressed genes (DEGs) previously identified in mTLE in humans by transcriptome analysis. We identified consensus DEGs among two independent mTLE transcriptome datasets and assigned them status as “lead target” if they (1) were involved in neuronal excitability, (2) were new in mTLE, and (3) were druggable. For this, we created a consensus DEG network in STRING and annotated it with information from the DISEASES database and the Target Central Resource Database (TCRD). Next, we attempted to validate lead targets using qPCR, immunohistochemistry, and Western blot on hippocampal and temporal lobe neocortical tissue from mTLE patients and non-epilepsy controls, respectively. Here we created a robust, unbiased list of 113 consensus DEGs starting from two lists of 3040 and 5523 mTLE significant DEGs, respectively, and identified five lead targets. Next, we showed that CACNB3, a voltage-gated Ca^2+^ channel subunit, was significantly regulated in mTLE at both mRNA and protein level. Considering the key role of Ca^2+^ currents in regulating neuronal excitability, this suggested a role for CACNB3 in seizure generation. This is the first time changes in CACNB3 expression have been associated with drug-resistant epilepsy in humans, and since efficient therapeutic strategies for the treatment of drug-resistant mTLE are lacking, our finding might represent a step toward designing such new treatment strategies.

**Supplementary Information:**

The online version contains supplementary material available at 10.1007/s12035-023-03426-4.

## Introduction

Mesial temporal lobe epilepsy (mTLE) is a circuit disorder characterized by an enduring predisposition to generate epileptic seizures from foci in the hippocampal formation, amygdala, and/or temporal neocortex [1]. The disorder is debilitating, persistent, and linked with major comorbidities and social consequences [2]. Differential gene expression levels identified in mTLE patients [3–5] may cause altered expression levels of specific proteins, such as ion channels, leading to generation of seizures [6]. Focal seizures account for approximately 60% of all adult cases, with TLE being the most common form causing focal seizure [7]. Despite intensive research, > 30% of mTLE patients are drug-resistant, which means that they do not achieve sustained seizure freedom with current antiseizure drugs (ASDs) [8]. The ASDs preclude seizure development by directly or indirectly controlling the ionic environment [9], but they do not cure epilepsy or block epileptogenesis. Due to the high unmet medical need in drug-resistant mTLE, there is a strong need for new drug targets to allow the development of better therapeutic strategies [3, 10].

We recently published a list of 3040 differentially expressed genes (DEGs) in mTLE [5]. With this study, we designed a drug-target discovery process that aims for disease modification rather than symptomatic relief of disease [10] by validation of new lead targets among the 3040 DEGs in human hippocampal and temporal lobe neocortical brain tissues on mRNA and protein level. However, 3040 DEGs are an overwhelming number to follow up on and easily result in biased selection (“cherry picking”) of genes already known by the investigator, thus overlooking new unknown targets [11].

Here, we reduced the list of 3040 mTLE significant DEGs to 113 DEGs using an unbiased bioinformatics approach and used systematic bioinformatics selection criteria to identify five lead targets. Next, we attempted to validate the selected lead targets using quantitative real-time PCR (qPCR), immunohistochemistry (IHC), and Western blotting performed on hippocampal and temporal lobe neocortical samples from mTLE patients and non-epilepsy control subjects. We show that CACNB3 (the β3 subunit of voltage-gated Ca^2+^ channels (VGCCs)) is significantly regulated in mTLE at mRNA and protein level. Alterations in VGCC expression levels and functionality are associated with several pathophysiological processes, such as epilepsy [12, 13], but this is the first time changes in CACNB3 expression have been associated with drug-resistant epilepsy in humans.

## Methods

### mTLE and Non-epilepsy Control Subject Tissues

Hippocampal and temporal lobe neocortical tissues from 17 mTLE patients were collected during brain surgery at the Departments of Neurology and Neurosurgery in Rigshospitalet, Copenhagen, as previously described [5]. An mTLE patient overview is presented in Table 1. Additional mTLE clinical data can be viewed in supporting information Table SI1. The contribution from clinical variation among mTLE patients onto the Kjær et al. dataset was assessed by principal component analysis (Fig SI3-16). Sixteen freshly frozen paired hippocampal and temporal lobe neocortical tissue samples and six hippocampal and six temporal lobe neocortical paraffin-embedded unpaired samples, respectively, from non-epilepsy control subjects were obtained from the UK Brain Banks Network–Medical Research Council (UKBBN) (including The Edinburg, The London Neurodegenerative Diseases, and The Oxford Brain Bank), The Human Brain Bank, Semmelweis University, and The Netherlands Brain Bank (population and sample characteristics can be viewed in Table SI2). Criteria for inclusion of non-epileptic control tissue were as closely matched as possible according to the following: (1) corresponding mean age at death (MAD) to the mTLE mean age at surgery (MAS), (2) matching sex distribution (SD) (females (F) and males (M)) among the groups, (3) no signs of autolysis upon histopathologic brain examination, and (4) no prior history of seizures (Table SI2).

**Table 1:** MTLE patient overview

Patient number	1	2	3	4	5	6	7	8	9	10	11	12	13	14	15	16	17
Duration of epilepsy (years)	13	26	47.5	31	6.5	10	7	35.5	38	43	33	6	2	7	25	9	45
ASDs at surgery	CBZ LEV	LTG LEV	CBZ LEV	LTG TPM	LTG ZNS BRI	LAC	LAC ZNS CLB	OXC CBZ CZP TPM	CBZ LEV LAC	LEV LAC	LAC LEV	CBZ LEV	LTG PER	LTG ZNS TPM	LEV OXC	LTG PER	PHT LEV TPM LAC
Gender	F	M	F	F	F	M	M	F	F	F	M	M	F	F	M	F	M
Age at surgery (years)	19	36	61	59	44	24	42	38	52	50	35	36	25	39	52	47	50
Age at epilepsy onset (years)	6	10	13.5	28	37.5	14	35	2.5	14	7	21	30	23	32	27	38	5
Focal onset aware* seizure frequency (*n*/month)	6	12	10	4	0	20	0	0	8	0	0	2	100	2	0	0	-
Focal onset impaired awareness* seizure frequency (*n*/month)	2	12	10	4	4.5	20	2	4.5	4	4	5	2	300	2	3.5	4.5	-
Focal to bilateral tonic clonic* seizure frequency (*n*/month)	˂0.1	˂0.1	˂0.1	˂0.1	˂0.1	0.1	0.1	˂0.1	1.5	˂0.1	0	1	0	0	1.5	0.1	0.5
Hippocampal pathology	HS	HS	HS	HS	No	HS	HS	HS	HS	HS	No	No	HS	No	HS	HS	HS
Psychiatric comorbidity	AD	No	De	No	No	No	De	Ps An	No	AD An	No	No	No	De	No	No	PT

*ASD*, anti-seizure drugs; *CBZ*, carbamazepine; *CLB*, clobazam; *CZP*, clonazepam; *BRI*, brivaracetam; *LAC*, lacosamide; *LEV*, levetiracetam; *LTG*, lamotrigine; *OXC*, oxcarbazepine; *PER*, perampanel; *PHT*, phenytoin; *TPM*, topiramate; *ZNS*, zonisamide; *M*, male; *F*, female; *n/month*, numbers per month; *HS*, hippocampal sclerosis (Wyler grades 3–4); *-*, unknown; *No*, no hippocampal pathology and/or psychiatric comorbidity; *AD*, attention deficit hyperactivity disorder; *De*, depression; *Ps*, psychosis; *An*, anxiety; *PT*, posttraumatic stress disorder; *, ILAE classifications 2017

The use of resected mTLE patient tissue, non-epilepsy control subject tissues from brain banks, and following procedures were approved by the local Ethical Committee in Copenhagen (H-2–2011-104). Written informed consent was obtained from all subjects prior to each surgery.

### Selection of DEGs by Consensus

As previously described, we identified 3040 DEGs between hippocampal and temporal lobe neocortical tissue in mTLE patients (FDR 5%) (GEO accession number: GSE134697) [5]. Another study by Guelfi et al. reported 5523 DEGs (FDR 5%) in temporal lobe neocortical tissue between a group of patients with mTLE and hippocampal sclerosis and a control group of non-epilepsy control subjects [4].

To select a subset of reliable DEGs from our study, we benchmarked our list of DEGs in relation to the genes reported by Guelfi et al., which allowed us to obtain a threshold that maximizes the overlap between the two datasets. The benchmarking strategy was as follows: we selected the top 500 DEGs from our study based on the S curve on the volcano plot (Fig SI18) and subsequently ranked them by their S score (Fig SI18). Next, we inspected the cumulative occurrence of these top 500 genes among the 5523 DEGs reported by Guelfi et al. [4], sorted by absolute log fold change (Log2FC), and then visually chose the first break of the curve as cut-off. The genes comprised on the cut-off list of DEGs were extracted, and the Log2FC associated to them according to the study by Kjær et al*.* was compared to the Log2FC reported by Guelfi et al. (Fig SI19). Genes that were regulated in opposite directions among studies were excluded from further analysis, while those that agreed on direction of regulation were named consensus DEGs.

### STRING Analysis

Cytoscape stringApp [14] was used to retrieve the STRING [15] network (version 10.5 using default settings) for the consensus DEGs (Fig SI20). Proteins in the STRING network, which were classified as kinases, G protein–coupled receptors (GPCRs), and ion channels, were annotated with target development level information from TCRD (http://juniper.health.unm.edu/tcrd/) [16] and with known associations to epilepsy from the DISEASES database [17].

### Selection Criteria for Lead Targets

Protein function was manually extracted from GeneCards [18] based on the “summaries of query gene” attribute (Table SI5), and STRING modules where more than half of the proteins are involved in excitatory and/or inhibitory neuronal mechanisms were manually selected. Proteins from all other modules were excluded despite being statistically significant. Proteins comprised in remaining modules were manually selected if their involvement in mTLE was new and their function was involved in excitatory and/or inhibitory neuronal mechanisms. Lead targets among short listed kinases, GPCRs, and ion channel were identified if they either/or (a) had an FDA-approved drug targeting them (TCRD classification, Tclin), (b) had a chemical compound modulating them (TCRD classification, Tchem), (c) had been biologically characterized (TCRD classification, Tbio), or was not biologically characterized (TCRD classification, Tdark) [16].

### qPCR

See “Method SI1” in supporting information for details.

### IHC

See “Method SI2” in supporting information for details.

### Western Blots

See “Method SI3” in supporting information for details.

## Results

**Fig. 1:**
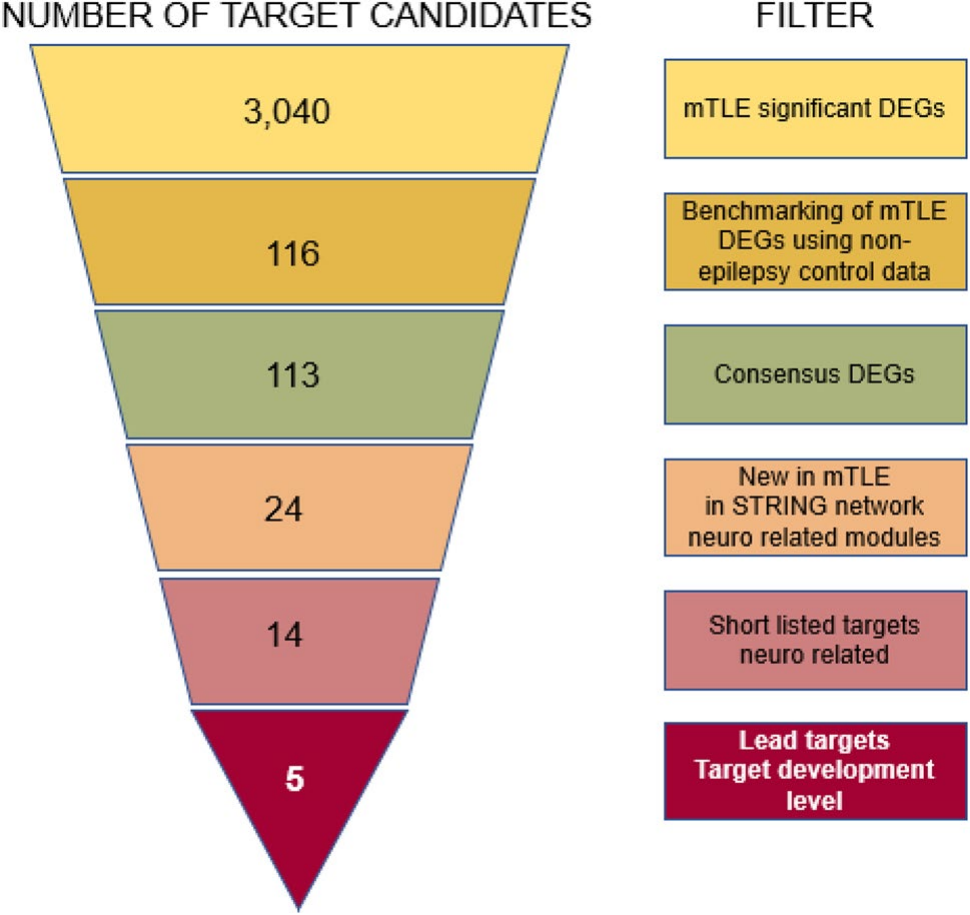
Lead target identification diagram. See text for details

In this paper, we first present a consensus list of DEGs based on two transcriptomics studies, perform a network analysis of these to identify neuro-related network modules, and use these to compile a list of lead targets that we follow up with experimental validation (Fig. 1).

### Selection of DEGs by Consensus

To identify a set of consensus genes that were consistently regulated among the Kjær et al. and the Guelfi et al. mTLE transcriptome datasets, we created a benchmark plot (Fig SI17A) that allowed us to obtain a threshold that maximized the overlap between the two datasets. We identified 116 DEGs in the Kjær et al. ranking among the 1485 DEGs in the Guelfi et al. ranking (Fig SI17A). Next, we extracted the list of 1485 DEGs, and the Log2FC associated to them according to the study by Kjær et al. was compared to the Log2FC reported by Guelfi et al. The comparison led to exclusion of three genes from further downstream analysis due to inconsistency in direction of gene regulation among the datasets (Fig SI19). The high agreement on DEG direction of regulation among the datasets (97.4%) indicates that the 113 DEGs represented on the consensus list are remarkably robust, since by chance we would expect only 50% agreement. The function of the 113 DEG gene products is presented in supporting information Table SI5.

### STRING and Druggability Analysis

The STRING network of the 113 consensus DEGs consists of 25 modules with at least two proteins each (Fig SI20). Twenty-four proteins had no interactions with any of the other proteins and were thus not considered in the subsequent analyses. Of the 25 modules, 11 had more than half of their proteins involved in excitatory and/or inhibitory neuronal mechanisms (Fig SI20; Table SI5). These modules comprised a total of 34 proteins of which 23 were new in terms of epilepsy and one was new in terms of mTLE. Ten of the 24 proteins were excluded due to their lack of involvement in excitatory and/or inhibitory neuronal mechanisms.

### Selection of Lead Targets

Among the 14 shortlisted proteins, CACNB3, KCNH5, KCNH7, HTR3B, and ZBTB20 were related to seizure generation in the brain [19–23], although their association to drug-resistant mTLE was largely unknown. The fact that these proteins are all ion channels also increased our confidence that they represented attractive seizure modulating drug targets in mTLE, since multiple currently marketed ASDs work by modulating ion channels. According to the TCRD [16, 24], the lead targets ranked as follows: KCNH5 and KCNH7 are even targets of FDA-approved drugs (Tclin), a chemical inhibitor of HTR3B exists (Tchem), while CACNB3 has been functionally characterized (Tbio) [16] (Fig SI17B; S21). We chose to include ZBTB20 as an exception, although it is neither a kinase, a GPCR, or an ion channel, because it was upregulated and thus constituted a possible desirable drug target [10], in addition to being linked to neurodevelopmental disorders [25].

### Validation of Lead Targets

**Fig. 2:**
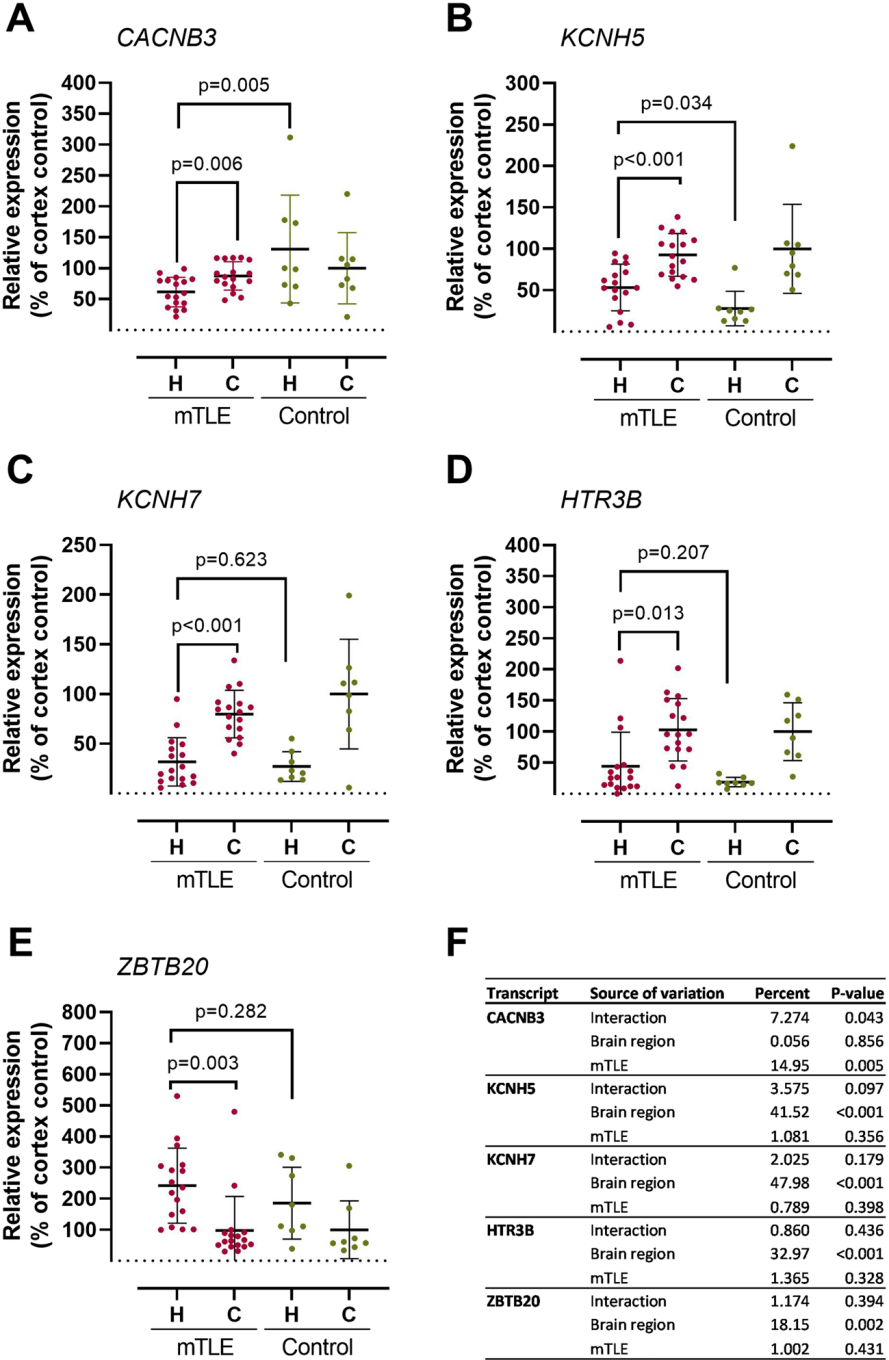
Relative mRNA expression as determined by qPCR of *CACNB3*, *KCNH5*, *KCNH7*, *HTR3B*, and *ZBTB20* in temporal lobe neocortices and hippocampus of mTLE patients or non-epilepsy control subjects confirm our earlier RNAseq-based findings and point to CACNB3 as a gene product of interest in mTLE. Panels **A**, **B**, **C**, **D**, and **E** show the relative transcript expression of *CACNB3*, *KCNH5*, *KCNH7*, *HTR3B*, and *ZBTB20*, respectively. Isolated RNA from 17 mTLE and 16 non-epilepsy subject (control) hippocampal and temporal lobe neocortical samples was analyzed by qPCR as described in the “Methods” section and the values obtained for temporal lobe neocortex for the non-epilepsy control subjects were normalized to 100%. Paired *t* tests were performed to compare the relative expression within the mTLE group, and unpaired *t* tests were performed to compare the relative expression in the hippocampus of the mTLE group vs. the non-epilepsy control subject group. The temporal lobe neocortex is designated “C,” and hippocampus is designated “H.” The mean and SD are indicated for all conditions and *p*-values of < 0.05 are considered significant. **F** Two-way analysis of variance was performed for all five transcripts and the contribution of brain region, mTLE, and the interaction between the two dependent variables to the total variance in the data are given in percent along with the computed *p*-values that are considered significant if < 0.05

qPCR on paired samples from 17 mTLE patients validated the previous transcriptome finding [5] that *CACNB3*, *KCNH5*, *KCNH7*, and *HTR3B* had lower expression levels and that *ZBTB20* had a higher expression level, respectively, in mTLE hippocampus compared to mTLE temporal lobe neocortex (Fig. 2A–E).

Next, we compared hippocampal expression level differences between mTLE and non-epilepsy control subjects since seizures most often originate from the amygdala-hippocampal complex [26]. *CACNB3* showed an expression level that was significantly lower in the mTLE hippocampus compared to the hippocampal control tissue while the opposite was observed for *KCNH5* (Fig. 2A, B). These results indicated that *CACNB3* expression level differences were related to disease while *KCNH5*, *KCNH7*, *HTR3B*, and *ZBTB20* expression level differences were related to brain region rather than disease. To gain further insight into the source of variation in the qPCR data, we conducted two-way analysis of variance. The result indicated that *CACNB3* regulation was significantly related to mTLE (source of variation, 14.95%; *p*-value, 0.005). In addition, the interaction between mTLE and brain region accounted for 7.27% of the total variation (*p*-value: 0.043), while the small percent for brain region had an insignificant *p*-value (source of variation, 0.056%; *p*-value, 0.856) (Fig. 2F).

Further, we calculated the mRNA expression ratios between the temporal lobe neocortex and the hippocampus for the two sample groups (Fig SI32). We did this to quantify the regional differences among mTLE patients and non-epilepsy control subjects. This analysis revealed that also the difference in expression of CACNB3 between the two brain regions was significantly affected by mTLE.

**Fig. 3:**
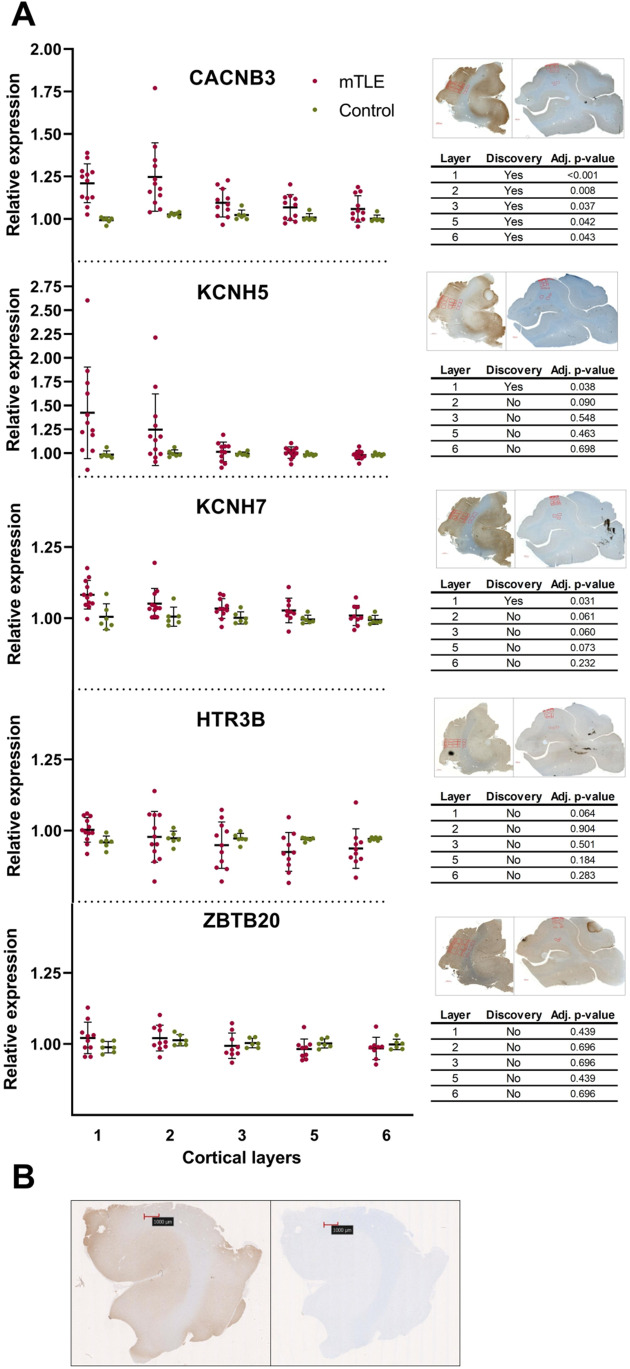
The relative protein expression levels as determined by immunohistochemical analysis of CACNB3, KCNH5, KCNH7, HTR3B, and ZBTB20 in layers 1, 2, 3, 5, and 6 of the temporal lobe neocortices of mTLE patients vs. non-epilepsy control subjects show that CACNB3 expression is higher in all layers for the mTLE patients. **A** Tissues from 14 mTLE patients and 12 non-epilepsy control subjects were analyzed by immunohistochemistry, and values represent relative expression levels as detailed in the “Methods” section. Multiple unpaired *t* tests with Welch’s correction and a false discovery rate of 5% were employed to test for differences between layers. The mean and SD are indicated for all conditions, and an adjusted *p*-value (adj.; the multiple test-corrected *p*-value) of < 0.05 is considered significant. Inserts show examples of representative marker-specific light field microscopy images (20 × magnification) of temporal lobe neocortical slices for a mTLE patient (left**)** and a non-epilepsy control subject (right). The red markings are measurements in resp. layers 1, 2, 3, 5, and 6 and white matter. **B** control slides of mTLE temporal lobe neocortex with CACNB3 staining (left) and without CACNB3 staining (right). Images of non-epilepsy control subject slides with and without CACNB3 staining are presented in SI35. All images may be viewed in large in supplemental information SI21-25, SI35, and SI36

To further explore whether our findings translated into results at the protein level, we conducted IHC analysis. Surprisingly, the deeper insight at protein level showed significant increased CACNB3 expression levels (immunoreactivity) in all temporal lobe neocortical layers compared to non-epilepsy control subjects, while KCNH5 and KCNH7 had showed increased expression levels in the first layer only (Fig. 3A; Fig SI21-25).

**Fig. 4:**
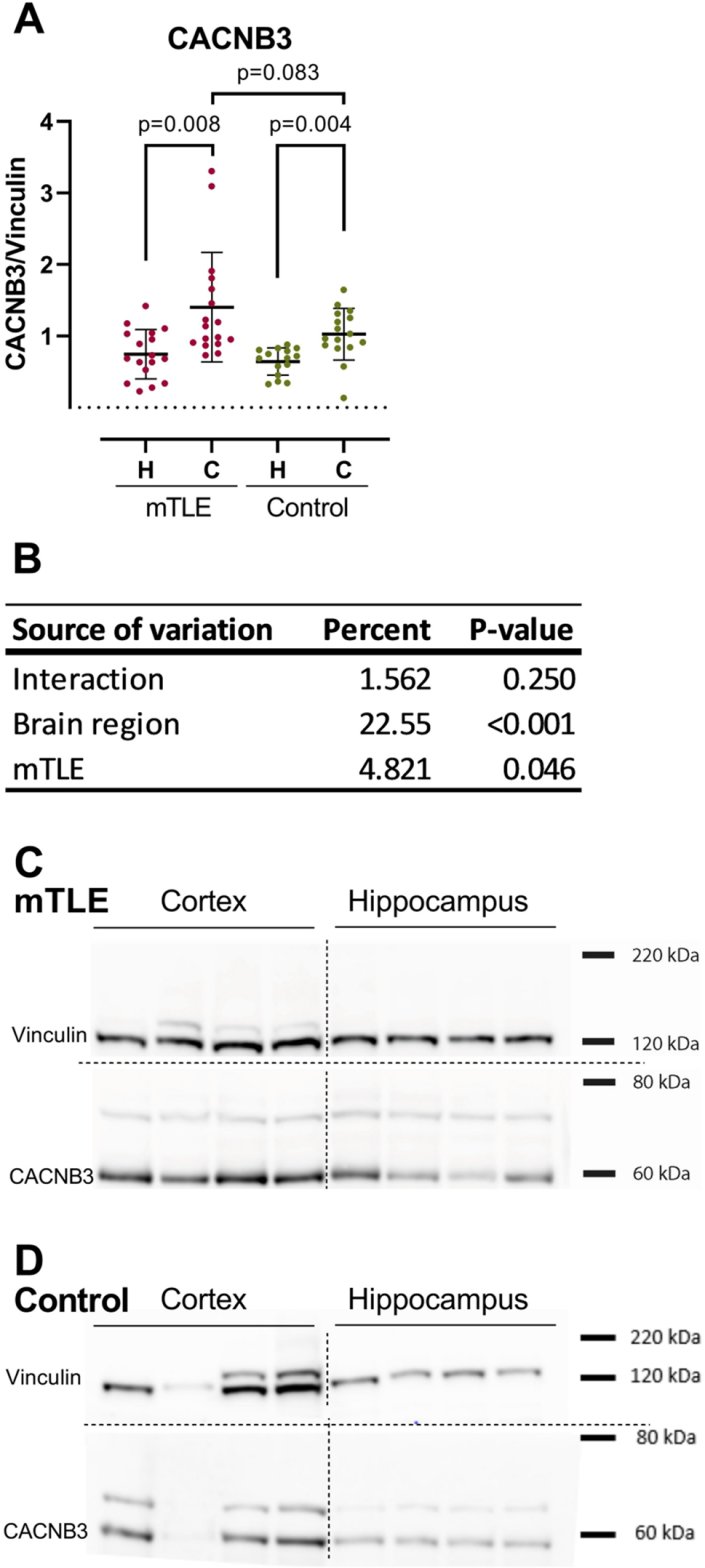
The relative protein expression levels as determined by Western blotting of CACNB3 in temporal lobe neocortex and hippocampus of mTLE patients or non-epilepsy control subjects show that expression of CACNB3 is higher in the temporal lobe neocortex relative to the hippocampus within the two groups but not between them. **A** Tissue from 17 mTLE patients and 16 control individuals were analyzed by Western blotting and values represent relative expression levels as detailed in the methods section. The values obtained for temporal lobe neocortex (designated “C”; hippocampus is designated “H”) for the non-epilepsy control subject group were normalized to 1. Multiple paired or unpaired *t* tests with Welch’s correction and a false discovery rate of 5% were employed to test for differences within and between the mTLE and the non-epilepsy control subject group, respectively. The mean and SD are indicated for all conditions, and *p*-values adjusted for multiple testing of < 0.05 are considered significant. **B** Two-way analysis of variance was performed to evaluate the contribution of brain region, mTLE, and the interaction between the two dependent variables to the total variance in the data; these are given in percent along with the computed *p*-values that are considered significant if < 0.05. **C** & **D** Representative blots showing four paired mTLE (C) and non-epilepsy control subject (D) samples, respectively, from temporal lobe neocortex (cortex) and hippocampus; vinculin is the loading control, and full blots showing all detected bands employed to construct panel C and D are presented in Fig SI33 and SI34

We detected no significant differences in KCNH5, KCNH7, HTR3B, CACNB3, and ZBTB20 hippocampal expression levels between mTLE and non-epilepsy control subjects (Fig SI26-31). Since the temporal lobe neocortical layer 1 only contains few neurons [27] (Fig SI1C), we reasoned that a significantly increased CACNB3 expression level affecting all molecular layers would have the greatest impact on brain function and therefore constituted the strongest lead target for mTLE involvement. Hence, we conducted Western blot on hippocampal and temporal lobe neocortical tissues, respectively, from mTLE and non-epilepsy control subjects, to possibly confirm the IHC CACNB3 finding. The Western blot result confirmed a significant decrease in CACNB3 expression level in mTLE hippocampus compared to temporal lobe neocortex (Fig. 4A; Table SI2) using a non-epilepsy cohort with an increased number of control subjects in the group compared to IHC analysis.

However, temporal lobe neocortical CACNB3 expression levels were also increased in non-epilepsy control subjects, questioning whether the mTLE CACNB3 increased expression levels were caused by brain region differences. Again, we conducted a two-way analysis of variance to clarify whether CACNB3 regulation was related to brain region or disease. This indicated that CACNB3 regulation was, indeed, disease-related (*p*-value: 0.046), with non-significant interaction (*p*-value: < 0.25; Fig. 4B) supporting our IHC finding. Western blot results also showed a tendency for higher temporal lobe neocortex CACNB3 expression in the mTLE group compared to non-epilepsy controls (Fig. 4A), although not statistically significant (*p*-value: 0.083). The fact that IHC is performed on specific neocortical layers while Western blotting is performed on homogenized tissue, which will dilute layer-specific differences, is a likely explanation to why the difference is only statistically significant in the former analysis.

To gain further insight into CACNB3’s likely involvement in seizure generation, we performed pathway analysis using the Reactome database (reactome.org). The result showed that CACNB3 is involved in the “presynaptic depolarization and Ca^2+^ channel opening” pathway. This suggests that altered CACNB3 expression levels may be involved in seizure generation by impairing normal neuronal presynaptic terminal function. Hence, changes in CACNB3 levels modify opening of voltage-gated Ca^2+^ channels which affect Ca^2+^ influx and neurotransmitter release and thereby influence neuronal excitability and seizure propensity.

### Summary of Main Findings

Using an unbiased bioinformatics approach, we reduced a list of 3040 mTLE significant DEGs to a robust list of 113 and identified *KCNH5*, *KCNH7*, *HTR3B*, *CACNB3*, and *ZBTB20* as lead targets. Among lead targets, we show consistent significant disease-related differences in expression level of CACNB3 in all lead target validation analyses performed, despite using different groups of non-epilepsy control tissues (Table 2). Changes in CACNB3 expression levels may thus be involved in mTLE pathophysiology and should be evaluated further for its molecular basis and value as putative new drug target in mTLE.

**Table 2:** Summary of main findings

Lead targets/analysis	CACNB3		KCNH5		KCNH7		HTR3B		ZBTB20	
RNA-Seq* mTLE temporal lobe neocortex vs. hippocampus (*p*-value)	↓ (˂0.0001)		↓ (˂0.0001)		↓ (˂0.0001)		↓ (˂0.0001)		↑ (˂0.0001)	
qPCR mTLE temporal lobe neocortex vs. hippocampus (*p*-value)	↓ (0.006)		↓ (˂0.0001)		↓ (˂0.0001)		↓ (0.013)		↑ (0.003)	
qPCR Hippocampal non-epilepsy control subjects vs. mTLE (*p*-value)	↓ (0.005)		↑ (0.036)		↑ NS (0.623)		↑ NS (0.207)		↑ NS (0.282)	
IHC Hippocampal** and temporal lobe neocortex*** non-epilepsy control subject vs. mTLE (adj. *p*-value)	Hippo	Cortex	Hippo	Cortex	Hippo	Cortex	Hippo	Cortex	Hippo	Cortex
	NS (> 0.800)	↑ All layers (˂0.041)	NS (> 0.604)	↑ 1^st^ layer (0.031) others (> 0.060)	NS (> 0.248)	↑ 1^st^ layer (0.031) others (> 0.061)	NS (> 0.925)	NS (> 0.063)	NS (> 0.574)	NS (> 0.438)
Western blot mTLE temporal lobe neocortex vs. hippocampus (*p*-value)	↓ (0.008)		ND		ND		ND		ND	
Western blot Non-epilepsy control subject temporal lobe neocortex vs. hippocampus (*p*-value)	↓ (0.004)		ND		ND		ND		ND	

*NS*, not significant; *ND*, no data; *Log2FC*, logarithmic fold change; * RNA sequencing data from Kjær et al. “*Transcriptome analysis in patients with temporal lobe epilepsy*” [5]; **, *p*-values represent both hilus and molecular layer; *** *p*-values represent cortical layers 1, 2, 3, 5, and 6 unless otherwise specified

Table 2 shows the directions and significance of mRNA and protein regulation of CACNB3, KCNH5, KCNH7, HTR3B, and ZBTB20 in hippocampus and temporal lobe neocortex in mTLE and non-epilepsy control subjects by the means of transcriptome analysis, qPCR, IHC, and Western blot analyses. Techniques and populations are listed in the first column. Lead targets are listed in the first row. The second row lists transcriptome results on direction of DEG regulation and the *p*-value expressed significance reported by Kjær et al. [5]. The third row lists mTLE hippocampal and temporal lobe neocortical qPCR results on direction of RNA regulation with a *p*-value expressed significance. The fourth row lists mTLE and non-epilepsy control subject hippocampal RNA expression level difference results from qPCR with the direction of regulation with a *p*-value expressed significance. The fourth row lists mTLE and non-epilepsy control subject hippocampal qPCR results on direction of RNA regulation with a *p*-value expressed significance. The fifth row lists mTLE and non-epilepsy control subject hippocampal and temporal lobe neocortical IHC results on protein regulation with a multiple testing adjusted *p*-value (adj. *p*-value) expressed significance. Significant *p*-values related to specific layers are specified, while non-significant findings are presented as most significant non-significant finding in all additional layers. The sixth row lists mTLE hippocampal and temporal lobe neocortical Western blot result on direction of protein level regulation with a multiple testing adjusted *p*-value expressed significance. The seventh row lists mTLE and non-epilepsy control subject hippocampal and temporal lobe neocortical Western blot result on direction of protein level regulation with a multiple testing adjusted *p*-value expressed significance. 

## Discussion

Drug resistance remains the leading cause of why more than 1/3 of epilepsy patients continue to have seizures despite best possible treatment [28], and identification of putative new drug targets is crucial to improve outcomes for patients with drug-resistant mTLE [29]. Several reports on transcriptome analysis have focused attention on DEGs as a source to identify new targetable molecular alterations in mTLE [4, 5, 30]. Here, we identify CACNB3 as a lead target for further exploration of the molecular basis of mTLE and its value as putative new drug target in mTLE.

CACNB3 is a cytosolic protein of VGCCs which is widely distributed throughout the human body [31] and plays a key role in the regulation of Ca^2+^ entry in excitable cells [31–33]. VGCC alterations are linked to pathophysiological processes such as epilepsy [12, 13], and CACNB3 promotes channel trafficking and regulates gating properties [34]. Four genes encode the β subunits (*CACNB1*-*CACNB4*), which all reportedly are expressed at similar levels in both pre- and postsynaptic hippocampal neurons, and all β subtypes are documented to enhance high voltage-activated calcium channel currents [31, 32, 34]. Increased intracellular Ca^2+^ concentration can alter neuronal excitability as a consequence of disrupted Ca^2+^ homeostasis, which may contribute to the generation of seizures [32, 35]. However, the functional roles of the β3 subunit are not fully clear, and it should be noted that CACNB3 has also been associated with IP_3_ receptor [33] and *N*-methyl-D-aspartate receptor (NMDAR) activity in the hippocampus [36]. CACNB3 has not previously been associated with drug resistance in epilepsy, but *CACNB3* expression level alterations were reported in studies addressing mental health conditions [37].

This validation study was based on our initial assumption from previously published RNA-seq results — that *CACNB3* is down-regulated in mTLE hippocampi compared to mTLE temporal lobe neocortices — because of disease [5]. The qPCR mRNA level results, which included hippocampal and temporal lobe neocortical tissue from both mTLE patients and non-epilepsy control subjects, supported that result (Fig. 2A). These findings cannot be explained by general cell death since the marker levels were normalized to *HPRT1* transcript levels which would change equally. However, they should be interpreted with the caveat that disrupted TLE tissue could have changes in cell type composition. Western blot protein level results confirmed CACNB3 downregulation in hippocampus compared to temporal lobe neocortex for the mTLE group, but not in the non-epilepsy control subjects (Fig. 4A), as will be discussed here. A study by Lin et al. showed that reduced VGCC function caused by a mutation affecting *CACNB4* significantly altered *CACNB3* expression levels in a lethargic *cacnb4*^*lh*^ mouse model of absence seizures when compared to controls [38]. Thus, *CACNB3* mRNA expression level alterations may contribute to seizures generation. However, while we found *CACNB3* expression levels significantly decreased in (1) mTLE hippocampus compared to temporal lobe neocortex (Fig. 2A) and (2) mTLE hippocampus compared to non-epilepsy control subjects (Fig. 2A), Lin et al. found them globally slightly increased throughout the brains of *cacnb4*^*lh*^ homozygote mice compared to controls [38]. Interestingly although not significant, we found *CACNB3* expression levels increased in hippocampus compared to those in temporal lobe neocortex in non-epilepsy control subjects (Fig. 2A), supporting our initial assumption that mTLE *CACNB3* expression level decrease in hippocampus compared to temporal lobe neocortex was related to disease. Lin et al. suggest that multiple classes of β subunit mRNAs segregate into distinct subcellular domains in hippocampal neurons [38], indicating an individual role of CACNB3 depending on cell and tissue type which could explain our divergent results to a certain degree. However, although the Lin et al. study showed that *CACNB3* alterations could be coupled to seizure generation, its upregulation was suggested as a compensatory effect on VGCC dysfunction mediating *CACNB4* downregulation [38].

Consistent with our mTLE mRNA findings, the deeper insight at protein level supported our assumption, since CACNB3 expression levels were decreased in mTLE hippocampus compared to temporal lobe neocortex (Fig. 4A). Surprisingly and in contrast to our mRNA level findings, Western blot results showed that CACNB3 expression levels were increased in temporal lobe neocortex compared to hippocampus in non-epilepsy control subjects (Fig. 2A), which did not support that *CABNB3* downregulated in mTLE hippocampus was disease related.

Despite an increased confidence that DEGs can be used for biological discovery at protein level [39], mRNA and protein expression level correlation seem poor across many studies [40, 41]. One reason is the many layers of regulatory processes known to cause deviating mRNA and protein expression levels (e.g., alternative polyadenylation and translation initiation) [42], and the pathophysiological mechanisms underlying mTLE are furthermore shown to give rise to an additional layer of gene expression regulation in epilepsy [43]. Thus, it is likely that our findings in the mTLE group are a consequence of disrupted regulatory processes. However, the result could also be caused by technical biases, since RNA and protein degrade differently (e.g., RNA is more easily degraded than protein, and different transcripts/proteins have different half-lives) [44, 45]. mRNA isolated from brain tissue stored for up to 160 min at room temperature upon collection was previously reported not to be degraded [46]. Given that all mTLE tissue was collected less than 3 min after resection [5], we found that the mTLE group unlikely was affected by technical biases to the same extent as non-epilepsy control subject tissues, which for ethical reasons have longer collection times. Differences in tissue handling, tissue collection method, etc. were furthermore unlikely to have influenced the mTLE group to the same extent as in the non-epilepsy group, from which the tissue arose from five different brain banks having five different procedures for tissue handling and collection.

IHC CACNB3 expression level results from temporal lobe neocortex were increased in all layers in mTLE compared to non-epilepsy control subjects, particularly in the upper neocortical layers (Fig. 3A). Combined with the recent finding that impairment of the upper cortical layers drives schizophrenia symptomatology [47], this may indicate that upper layer cortical networks are a hotspot for neurodevelopmental disorders and the most vulnerable for impairments. Our IHC finding is also consistent with the near-significant result from Western blot analysis (Fig. 4A), and since both analyses represent different non-epilepsy control subject tissue samples, it gave us an enhanced confidence that the increased CACNB3 expression levels in mTLE temporal lobe neocortex were a true biological finding. In accordance with our findings, N’Gouemo et al. reported increased CACNB3 expression levels in genetically epilepsy-prone rats (GEPR) [48], while Lie et al. reported an increased distribution of CACNB1 and CACNB2 in TLE hippocampus compared to controls [49]. The latter likely led to enhanced currents carried by VGCCs, which thereby increased synaptic excitability and triggered epileptic seizures [49]. Thus, changes in CACNB3 expression levels may be associated with changes in excitability, although it remains to be further explored to what extent these changes contribute to increased excitability in mTLE. However, N’Gouemo et al. studied the inferior colliculus (the consensus site for seizure initiation in GEPR) neurons compared to control rats [48] which is not the consensus site for seizure initiation in mTLE [26], questioning the comparability of the studies. Since seizures most often originate in the amygdala-hippocampal complex in mTLE patients [26], it makes sense that hippocampus is most affected by disease and thus is the best tissue to study when addressing epilepsy-associated pathology. On the other hand, hippocampal tissue in mTLE patients usually shows severe degeneration (Table 1), harboring the risk that mTLE hippocampal studies reflect degeneration and not epileptogenic effects. The progressive nature of mTLE (increasingly larger parts of the brain is affected by mTLE [26]) furthermore makes it likely that increased CACNB3 expression levels may also be coupled to seizure generation originating from the temporal lobe neocortex [26]. However, as Lin et al. points out, it is not clear whether CACNB3 expression alterations are causal or compensatory in relation to mTLE pathophysiology [38], emphasizing the relevance of further investigating of the role of CACNB3 in mTLE temporal lobe neocortical pathophysiology and as putative new drug target in mTLE.

It should certainly be feasible to design drugs that can modulate CACNB3 activity. One putative approach would be to design a proteolysis targeting chimera (PROTAC) type drug that could modify the presence of functional CACNB3 protein by regulating proteasomal degradation [50]. Within the existing class of ion channel–targeting drugs, this approach would be novel since a PROTAC targeting CACNB3 would modulate channel activity without blocking the channel itself. Given that we are looking at tissue from patients who already have the disease, there is inherently no way to tell whether regulated CACNB3 levels are a cause or a consequence of the disease. This would be the case even if we could exclude it being an effect of degeneration of specific cell types or of glial activation. However, as the aim of our study is to identify putative novel drug targets for the disease — and many good drug targets are not causal — establishing causality is not essential to our study. Considering the success of gabapentinoids that block α2δ-1 of VGCCs thereby reducing Ca^2+^ influx and neurotransmitter release [51, 52], it is clear that targeting auxiliary subunits of ion channels can exert significant clinical effect. However, to develop CACNB3 as a drug target requires more than a disease-modifying role for CACNB3 and a feasible medicinal chemistry strategy, as discussed by Gashaw et al. [10]. For instance, while CACNB3 is primarily expressed in the brain, it is expressed and serves key roles in several other tissues [31–33, 53]. Thus, developing CACNB3 as a drug target might require a brain-selective drug delivery strategy as well. In summary, much work is needed before CACNB3 can be considered further as a putative novel drug target.

## Conclusion

We unbiasedly reduced our initial list of 3040 significant mTLE DEGs down to 113 using bioinformatics and identified *CACNB3, KCNH5*, *KCNH7*, *HTR3B*, and *ZBTB20* as lead targets in mTLE using a systematic bioinformatics strategy. qPCR, IHC, and Western blot results on mTLE and non-epilepsy control subject tissues indicated that CACNB3 expression level alterations were likely to be caused by disease. Thus, here we provide a groundwork understanding of CACNB3 expression alterations in mTLE, suggest its possible involvement in mTLE pathophysiology, and reflect on its value as a putative new drug target. Given the diverse roles suggested to be mediated by CACNB3, its functional biological roles and putative association to mTLE are not well understood. Consequently, we encourage follow-up studies addressing whether *CACNB3* downregulation in hippocampus and CACNB3 upregulation in temporal neocortex is critically involved in mTLE and whether modulation of CACNB3 with drugs is likely to have a positive therapeutic effect. The dataset of Kjær et al. is well suited for further analyses of targets in mTLE, including neuroinflammation and cell adhesion.

## Supplementary Information

Below is the link to the electronic supplementary material.

Supplementary file1 (PDF 2414 KB)

## Data Availability

The raw expression datasets analyzed during the current study are available on the Gene Expression Omnibus (GEO) repository with the accession codes GSE134697 (https://www.ncbi.nlm.nih.gov/geo/query/acc.cgi?acc=GSE134697) and GSE46706 (https://www.ncbi.nlm.nih.gov/geo/query/acc.cgi), respectively.
