# Supplementary material for: Differential Expression of the β3 Subunit of Voltage-Gated Ca2+ Channel in Mesial Temporal Lobe Epilepsy
Source: Mol Neurobiol. 2023 Jun 21;60(10):5755–69. doi: 10.1007/s12035-023-03426-4 (PMC10471638; doi:10.1007/s12035-023-03426-4)
Supplement: Supplementary file 1 — Supplementary file1 (PDF 2414 KB) [file 12035_2023_3426_MOESM1_ESM.pdf]

Supporting Information

Differential expression of the β3 subunit of voltage gated Ca^{2+}channel in mesial temporal lobe epilepsy

Christina Kjær[1,2], Oana Palasca[3], Guido Barzaghi[2,*1], Lasse K Bak[2,4], Rúna KJ Durhuus[2,*2], Emil Jakobsen[2,*3], Louise Pedersen[1,4], Emil D Bartels[4,7], David PD Woldbye[5], Lars H Pinborg[6], Lars Juhl Jensen[3]

[1]Department of Technology, Faculty of Health and Technology, University College Copenhagen, DK-2200 Copenhagen, Denmark

[2]Department of Drug Design and Pharmacology, Faculty of Health and Medical Sciences, University of Copenhagen, 2100, Copenhagen, Denmark

[3]Disease Systems Biology Program, Novo Nordisk Foundation Center for Protein Research, Faculty of Health and Medical Sciences, University of Copenhagen

[4]Dept. of Clinical Biochemistry, Rigshospitalet, DK-2600, Copenhagen, Denmark

[5]Department of Neuroscience, University of Copenhagen, DK-2200 Copenhagen, Denmark

[6]Epilepsy Clinic & Neurobiology Research Unit, Copenhagen University Hospital, University of Copenhagen, DK-2100 Copenhagen, Denmark

[7]Department of Clinical Medicine, University of Copenhagen, Copenhagen, Denmark

Corresponding author contact details: Christina Kjær, University College Copenhagen, Department of Technology, Biomedical Laboratory Science, Sigurdsgade 26, 1st 2200 København N, Denmark. E-mail: chkj@kp.dk

---

*1 European Molecular Biology Laboratory (EMBL), Genome Biology Unit, Heidelberg, Germany. Faculty of Biosciences, Collaboration for Joint PhD Degree between EMBL and Heidelberg University, Heidelberg, Germany

*2 Specific Pharma a/s, Borgmester Christiansens Gade 40, 2450 Copenhagen SV, Denmark

*3 Takeda Pharma A/S, Delta Park 45, 2665 Vallensbaek Strand, Denmark

Table SI1 - Extended mTLE patient overview

Patient number	1	2	3	4	5	6	7	8	9	10	11	12	13	14	15	16	17
Known etiology	No	INF	No	No	No	No	No	No	No	No	No	No	No	No	No	No	No
Impaired language ability*	Yes	Yes	Yes	No	No	No	Yes	No	Yes	No	Yes	Yes	Yes	Yes	No	Yes	Yes
Impaired memory ability*	No	No	Yes	Yes	No	No	Yes	No	Yes	No	Yes	Yes	Yes	Yes	Yes	Yes	Yes
Learning disability*	Yes	No	No	No	No	No	No	No	No	No	No	No	No	No	Yes	No	Yes
Tobacco use preoperative** (items/day)	0	0	7.5	15	0	0	7	0	6	0	0	0	10	0	30	20	0
Alcohol use preoperative** (items/week, 1 item = 33cl.)	0	0	4.5	0	0	0	14	2	0	0	40	3	3	0	0	5	0
Drug abuse (times/week)**	0	0	0	0	0	0	0	0	0	0	1	0	0	0	0	0	0
ASDs previously	OXC<br>LAC	-	CBZ	-	LEV<br>VPA<br>LAC<br>PER	TPM<br>LEV<br>ZNS<br>VPA	OXC<br>LEV	-	LTG<br>VPA<br>CLB	LTG<br>VPA	-	-	CLO	VPA<br>CLO<br>PER<br>LEV	CBZ<br>PB<br>ZNS<br>TPM	-	-

INF, infectious; CBZ, carbamazepine; CLB, clobazam; CLO, clonazepam; LAC, lacosamide; LEV, levetiracetam; LTG, lamotrigine; OXC, oxcarbazepine; PB, phenobarbital; PER, perampanel; TPM, topiramate; VPA, valproate; ZNS, zonisamide; -, unknown; *, diagnose based on neuropsychological testing; **, self reported by patients

Table SI2. Population characteristics of non-epilepsy control subjects

Subject	Sex (M, F)	Age at Death	PMD (hours)	Analysis Type	Cause of death/ Clinical diagnosis
**1**	F	45	40	WB	Suicide by hanging, paranoid schizophrenia
**2**	M	38	36	WB	Ischaemic heart disease, coronary artery atherosclerosis, type 2 diabetes mellitus
**3**	M	51	23	WB	Coronary artery disease
**4**	F	42	48	WB	Metastatic pancreatic carcinoma
**5**	M	40	40	WB	Liver, renal & respiratory failures
**6**	F	43	43	WB	Pneumonia; severe obesity
**7**	F	47	27	WB	Endstage of renal failure, diabetic nephropathy, diabetes mellitus, small bowel perforation
**8**	F	51	33	WB	Lung cancer
**9**	M	49	2	qPCR, WB	Acute cardiac insufficiency, coronary sclerosis
**10**	M	51	1	qPCR, WB	Cardiorespiratory insufficiency, sudden death
**11**	M	66	4.5	qPCR, WB	Cardiovascular-pulmonary insufficiency, acute lymphoid leukemia
**12**	M	27	8	qPCR, WB	Pneumonia
**13**	M	54	2	qPCR, WB	Acute myocardial infarction, sudden death
**14**	F	26	6.5	qPCR, WB	Acute myocardial infarction
**15**	M	53	2	qPCR, WB	Acute myocardial infarction, sudden death
**16**	F	72	1	qPCR, WB	Pulmonary embolism
**17**	M	57	4.5	IHC	Myocardial infarction
**18**	M	49	5.5	IHC	Pulmonary nocardiosis with a brain abscess
**19**	F	54	12.45	IHC	Traffic accident
**20**	F	32	< 41.00	IHC	Myocardial infarction
**21**	M	38	10.45	IHC	M. Wegener; aluminium intoxication
**22**	F	47	4	IHC	Respiratory failure due to pleuritis and extensive lymphangitis carcinomatosis with a "trapped-lung"
**23**	F	50	4	IHC	Metastasized large cell bronchocarcinoma
**24**	M	49	6.15	IHC	Euthanasia
**25**	M	55	7.30	IHC	Euthanasia with esophageal cancer

**26**	M	55	7.15	IHC	Intestinal ischemia by thrombosis of the a. mesenterica superior
**27**	F	60	5.30	IHC	Euthanasia
**28**	F	55	7.30	IHC	Euthanasia

M: Male; F: Female; PMD: Postmortem delay; qPCR: quantitative polymerase chain reaction; WB: western blot; IHC: immunohistochemistry; Subject 1-2: paired fresh frozen hippocampal and temporal lobe neocortex from case BBN_22618 and BBN_21396 (BBN number is a unique Medical Research Council database number) from Edinburg Brain Bank; Subject 3-4: paired fresh frozen hippocampal and temporal lobe neocortex from Oxford Brain Bank case BBN_11051 and BBN_10601; Subject 5-8: paired fresh frozen hippocampal and temporal lobe neocortex from Kings College London Brain Bank case BBN_4193, BBN002.30139, BBN_16253 and BBN_15790; Subject 9-16: paired fresh frozen hippocampal and temporal lobe neocortex from The Human Brain Bank, Semmelweis University; Subject 17-28: un-paired paraffin embedded hippocampal (six pieces) and temporal lobe neocortex (six pieces) from The Netherlands Brain Bank

Table SI3 – Primers uses for qPCR

Gene	Forward primer (sequence 5′-3′)	Reverse primer (sequence 5′-3′)
HPRT1	CATTATGCTGAGGATTTGGAAAGG	CTTGAGCACACAGAGGGCTACA
	Amplicon length: 129	
HTR3B	CCCTACCTCTAAGTGCCATCTG	AGAGACCACCTGGATGGGCTTA
	Amplicon length: 140	
ZBTB20	GACTTTCACCGCCAAACAGA	CTTTAAGGAGAAGGAGCGCC
	Amplicon length: 100	
CACNB3	TTGGACGCTGACACCATCAACC	AGCGAATGAGACGCTGGAGTAC
	Amplicon length: 112	
KCNH5	GTCAGGCAAACTTTTGACAACTAC	ATCCTTGAAAGTACACAGGAACAAG
	Amplicon length: 150	
KCNH7	ATACATTTCTGGGGACCATCAT	GAGAAACCAGTCATCTCACAGAAC
	Amplicon length: 130	

Table SI4 - Immunohistochemical antibodies used in the study

Antibody	Clone (company)	Dilution	Antigen Retrieval	Detection system
CACNB3	CAB3(PA5-77301) (Invitrogen)	1: 100	Heat Induced Epitop Retrievel/ ULTRA CC1 (pH 9)	Zeiss Axio Scan Z1 microscope, (Ziess, Germany)
KCNH5	hEAG2(ab224482) (Abcam)	1:100	Heat Induced Epitop Retrievel/ ULTRA CC1 (pH 9)	Zeiss Axio Scan Z1 microscope, (Ziess, Germany)
KCNH7	hERG-3(PA5-53713) (Invitrogen)	1:100	Heat Induced Epitop Retrievel/ ULTRA CC1 (pH 9)	Zeiss Axio Scan Z1 microscope, (Ziess, Germany)
HTR3B	5-HT3B(ab39629) (Abcam)	1:1000	Heat Induced Epitop Retrievel/ ULTRA CC1 (pH 9)	Zeiss Axio Scan Z1 microscope, (Ziess, Germany)
ZBTB20	Zinc finger protein 288(ab127702) (Abcam)	1:75	Heat Induced Epitop Retrievel/ ULTRA CC1 (pH 9)	Zeiss Axio Scan Z1 microscope, (Ziess, Germany)

Table SI5 - Direction and level of regulation and function of the 113 mTLE significant differential expressed genes (DEGs)

DEG	Log2FC	Gene function
**ASB2**	-2.57	Plays a role in retinoic acid-induced growth inhibition and differentiation of myeloid leukemia cells
**TIAM2**	-1.24	Involved in lamellipodial formation and advancement of the growth cone of embryonic hippocampal neurons. Promotes migration of neurons in the cerebral cortex
**PDP1**	-1.44	An enzyme located in the mitochondrial matrix
**NUAK1**	-0.96	Serine/threonine-protein kinase involved in various processes such as cell adhesion
**MT1X**	1.11	May be involved in FAM168A anti-apoptotic signaling
**CBLN2**	-1.83	May play role in synaptogenesis induction
**PKNOX2**	-1.09	A transcription factor. Plays fundamental roles in cell proliferation and differentiation
**TBR1**	-1.48	Transcriptional repressor involved in multiple aspects of cortical development, including neuronal migration, laminar and areal-identity, and axonal projection
**RIMS3**	-1.88	Regulates synaptic membrane exocytosis
**RHBDF2**	0.99	Regulates ADAM17 protease and thereby plays a role in sleep, cell survival, proliferation, migration, and inflammation
**KCNQ5**	-1.58	A member of the KCNQ potassium channel gene family that is differentially expressed in subregions of the brain and in skeletal muscle
**TJP2**	1.42	Functions as a component of the tight junction barrier in epithelial and endothelial cells and is necessary for proper assembly of tight junctions
**CHML**	-1.05	Substrate-binding subunit (component A) of the Rab geranylgeranyltransferase (GGTase) complex
**NRGN**	-1.32	A postsynaptic protein kinase substrate that binds calmodulin in the absence of calcium
**FAM189A2**	1.09	In family with Sequence Similarity 189 Member A2
**LPCAT2**	1.00	May function in membrane biogenesis and production of platelet-activating factor in inflammatory cells

**CPNE9**	-2.30	May play a role in calcium-mediated intracellular processes. Involved in positive regulation of dendrite extension.
**TMEM155**	-1.71	Transmembrane protein
**SLC6A7**	-1.51	A member of the gamma-aminobutyric acid (GABA) neurotransmitter gene family and encodes a high-affinity mammalian brain L-proline transporter protein
**ID3**	1.15	A helix-loop-helix (HLH) protein that can form heterodimers with other HLH proteins
**CDH7**	-0.84	A calcium-dependent cell adhesion protein
**EPHX4**	-1.66	A hydrolase
**SLC14A1**	2.11	Involved in urea transport. Plays an important role in the kidney medulla collecting ducts
**LNX1**	-1.45	An E3 ubiquitin-protein ligase involved in signal transduction and protein interactions
**C4B**	1.48	A part of the classical complement activation pathway
**CHST6**	1.59	An enzyme that catalyzes the transfer of a sulfate group to the GlcNAc residues of keratan
**SERPINE1**	2.02	A primary inhibitor of tissue-type plasminogen activator and urokinase-type plasminogen activator
**RIMS2**	-1.14	Important for normal neurotransmitter release
**CAMKK2**	-1.75	Influences signaling cascades involved with learning and memory, neuronal differentiation and migration, neurite outgrowth, and synapse formation
**DDR1**	1.06	A receptor tyrosine kinase that plays a key role in the communication of cells with their microenvironment
**TLR3**	0.88	A member of the Toll-like receptor family which plays a fundamental role in pathogen recognition and activation of innate immunity
**ITGAM**	0.79	A part of the integrin alpha M chain
**HTR3B**	-3.23	Serotonin receptor sub-unit B3. Mediates fast depolarizing responses in neurons after activation
**POU6F**	-1.17	Probable transcription factor likely to be involved in early steps in the differentiation of amacrine and ganglion cells

**TYRP1**	-2.85	Involved in melanin and albinism
**RGS1**	2.46	Regulates G protein-coupled receptor signaling cascades
**TMEM196**	-1.92	A transmembrane Protein 196
**CH25H**	2.36	Involved in cholesterol and lipid metabolism
**TNC**	2.47	Extracellular matrix protein implicated in guidance of migrating neurons as well as axons during development, synaptic plasticity as well as neuronal regeneration
**IL1RAPL2**	-2.31	An interleukin involved in immune responses, inflammatory reactions, and hematopoiesis. It is associated with X-linked non-syndromic cognitive disability
**SRPX**	1.83	May be involved in phagocytosis during disk shedding, cell adhesion to cells other than the pigment epithelium or signal transduction
**CD38**	1.75	Type II transmembrane glycoprotein that synthesizes and hydrolyzes cyclic adenosine 5'-diphosphate-ribose. Is an intracellular calcium ion mobilizing messenger
**SRGN**	0.88	A hematopoietic cell granule proteoglycan. Plays a role in formation of mast cell secretory granules and mediates storage of various compounds in secretory vesicles
**MKX**	-1.61	May play a role in cell adhesion
**RASD2**	-1.38	An activator of mechanistic target of rapamycin 1 (mTOR1)
**GEM**	1.23	Could be a regulatory protein, possibly participating in receptor-mediated signal transduction at the plasma membrane
**RHEBL1**	-1.61	Binds GTP and exhibits intrinsic GTPase activity. Promotes signal transduction through MTOR
**MGAT5B**	-2.16	Plays an active role in modulating integrin and laminin-dependent adhesion and migration of neuronal cells via its activity in the O-mannosyl glycan pathway
**GALNT15**	-0.96	Catalyzes the initial reaction in O-linked oligosaccharide biosynthesis
**CD44**	2.15	A cell-surface glycoprotein involved in cell-cell interactions, cell adhesion and migration
**CRABP1**	-2.69	Retinoic acid-mediated differentiation and proliferation processes
**HECW1**	-1.48	A ubiquitin-protein ligase

**ITGB4**	1.67	Integrins mediate cell-matrix or cell-cell adhesion. and transduced signals that regulate gene expression and cell growth
**TEX29**	-1.36	Testis-Expressed Protein 29
**SMPX**	-2.7	Plays a role in the regulatory network through which muscle cells coordinate their structural and functional states during growth. adaptation. and repair
**TMEM132D**	-1.35	May serve as a cell-surface marker for oligodendrocyte differentiation
**LPAR4**	1.51	Member of a family of receptors that bind purine and pyrimidine nucleotides and are coupled to G proteins
**PLCB4**	-0.85	Has a role in retina signal transduction
**GIG25**	3.66	A plasma protease inhibitor and member of the serine protease inhibitor class
**CD74**	1.06	An important chaperone that regulates antigen presentation for immune response
**NFKBIA**	0.95	Inhibits the activity of dimeric NF-kappa-B/REL complexes by trapping REL dimers in the cytoplasm through masking of their nuclear localization signals
**EPN3**	-1.69	Involved in cell junction dynamics
**ITPKA**	-1.67	Important in cellular signaling
**HLA-DPA1**	1.09	Plays a central role in the immune system by presenting peptides derived from extracellular proteins
**FPR1**	1.11	Mediates the response of phagocytic cells to invasion of the host by microorganisms and is important in host defense and inflammation
**KCNH7**	-1.76	Voltage-gated potassium (Kv) channel
**LDB2**	-1.47	Binds to the LIM domain of a wide variety of LIM domain-containing transcription factors
**NGEF**	-1.54	Plays a role in axon guidance regulating ephrin-induced growth cone collapse and dendritic spine morphogenesis
**RXFP1**	-1.91	Plays a critical role in sperm motility. pregnancy and parturition as a receptor for the protein hormone relaxin
**IPCEF1**	-1.30	Enhances the promotion of guanine-nucleotide exchange by PSCD2 on ARF6 in a concentration-dependent manner

**IL1B**	2.03	Potent pro-inflammatory cytokine
**FBLN7**	-1.43	Adhesion molecule that interacts with extracellular matrix molecules in developing teeth well as in dentin formation
**CACNA1G**	-1.59	Voltage-sensitive calcium channels (VSCC) mediate the entry of calcium ions into excitable cells
**MTCL1**	-1.07	Plays a role in the development and maintenance of non-centrosomal microtubule bundles at the lateral membrane in polarized epithelial cells
**RORB**	-1.39	A member of the NR1 subfamily of nuclear hormone receptors
**ATRNL1**	-1.43	May play a role in melanocortin signaling pathways that regulate energy homeostasis
**ZNF697**	-1.49	A zinc-finger. May be involved in transcriptional regulation
**PDK4**	1.13	A mitochondrial protein with a histidine kinase domain
**TNNT2**	-2.36	Regulates muscle contraction in response to alterations in intracellular calcium ion concentration
**KCNH5**	-1.62	Pore-forming (alpha) subunit of voltage-gated potassium channel. Elicits a non-inactivating outward rectifying current
**TRHDE**	-1.51	An extracellular peptidase that specifically cleaves and inactivates the neuropeptide thyrotropin-releasing hormone
**TPBG**	-2.10	A leucine-rich transmembrane glycoprotein that may be involved in cell adhesion
**CXCR4**	1.22	A CXC chemokine receptor specific for stromal cell-derived factor-1
**SATB2**	-1.34	A DNA binding protein that specifically binds nuclear matrix attachment regions
**FCGR2A**	0.96	Family of immunoglobulin Fc receptor genes found on the surface of many immune response cells
**HLA-DOA**	1.28	HLA class II alpha chain paralogues.
**ITPR1**	-1.45	Intracellular channel that mediates calcium release from the endoplasmic reticulum following stimulation by inositol 1,4,5-trisphosphate
**TESPA1**	-3.4	T-cell and mitochondria involvement
**EPHB6**	-1.55	Can influence cell adhesion and migration
**CYP4F3**	1.72	Member of cytochrome P450 superfamily
**KRT5**	-2	Keratin

**CABP1**	-1.43	Modulates calcium-dependent activity of inositol 1,4,5-triphosphate receptors
**GLRA2**	-1.52	A glycine receptor alpha subunit
**KCNS1**	-2.31	Potassium channel subunit that does not form functional channels by itself. Can form functional heterotetrameric channels with KCNB1 and KCNB2
**KCNS2**	-1.89	Potassium channel subunit that does not form functional channels by itself
**KCNV1**	-1.73	Potassium channel subunit that does not form functional channels by itself
**KCNH1**	-1.02	Pore-forming (alpha) subunit of a voltage gated delayed rectifier potassium channel
**SGK1**	1.48	A serine/threonine protein kinase that plays an important role in cellular stress response
**IER3**	1.29	Protection of cells from Fas- or tumor necrosis factor type alpha-induced apoptosis
**DNAJC5G**	-2.42	DnaJ Heat Shock Protein Family (Hsp40) Member C5 Gamma
**ZFP36**	2.05	Zinc-finger RNA-binding protein that destabilizes several cytoplasmic AU-rich element (ARE)-containing mRNA transcripts
**RGS4**	-1.57	Inhibits signal transduction by increasing the GTPase activity of G protein alpha subunits
**FOS**	1.91	Nuclear phosphoprotein which forms a tight but non-covalently linked complex with the JUN/AP-1 transcription factor
**C3AR1**	1.15	Receptor for the chemotactic and inflammatory peptide anaphylatoxin C3a
**PRKAR2B**	-1.02	Regulatory subunit of the cAMP-dependent protein kinases involved in cAMP signaling in cells
**EGR2**	1.76	A transcription factor with three tandem C2H2-type zinc fingers
**SELL**	1.42	A cell surface adhesion molecule that belongs to a family of adhesion/homing receptors
**FCER1G**	0.90	High affinity IgE receptor is a key molecule involved in allergic reactions
**CACNB3**	-1.39	A regulatory beta subunit of the voltage-dependent calcium channel
**TMEM119**	0.94	Plays an important role in bone formation and normal bone mineralization

**PDGFD**	-1.54	Platelet-derived growth factor family. plays an essential role in the regulation of embryonic development
**ZBTB20**	0.86	Dendritic cell derived BTB/POZ zinc finger (DPZF). Acts as a transcriptional repressor and plays a role in many processes including neurogenesis
**LILRB4**	0.97	A leukocyte immunoglobulin-like receptor (LIR) family member

Log2FC, logarithmic fold change of normalized RNA-Seq read counts between mTLE hippocampus and temporal lobe neocortex; Gene function, taken from genecards.org “Summaries of genes”.

Method S1

qPCR

RNA was extracted from 17 mTLE (MAS: 42 years; SD: 10 F & 7 M, Table 1) and 8 non-epilepsy control subject (MAD: 50 years; SD: 6 F & 2 M, Table SI2) hippocampal and temporal lobe neocortical flash frozen biopsies (Table SI2) using AllPrep DNA/RNA/Protein Extraction Kit (Qiagen) according to the manufacturer's instructions. The integrity of the purified RNA was analyzed using an RNA Nano LabChip (Agilent Technologies). 250 ng RNA from each sample was used for cDNA synthesis according to the manufacturer's instructions (High-Capacity cDNA Reverse Transcription kit, Applied Biosystems™, Foster City, USA). qPCR was performed with Quantstudio™ 12K Flex Real-Time PCR System® (Applied Biosystem) using the SYBR-green reagent (Applied Biosystem, cat.no: 4385617). Primer sequences are described in Table SI3. The relation between the time point of the log-linear increase of the fluorescence signal and the relative expression level of each transcript in each tissue biopsy was determined by parallel analyses of dilutions of pooled brain cDNA. Gene expression of target genes was normalized to the content of the house keeping gene HPRT. The expression of HPRT was similar in all groups regardless of anatomical localization and disease status.

Method S2

IHC

Hippocampal and temporal lobe neocortex resection tissue (50-200 mg pieces) obtained after surgery of 14 patients (MAS: 44 years; SD: 8 F & 6 M, Table 1) with drug-resistant mTLE at Copenhagen University Hospital were fixed in 10% formalin and paraffin-embedded. Post-surgical evaluation of the hippocampus was performed by a pathologist at Copenhagen University Hospital, and diagnosis for hippocampal sclerosis was determined according to ILAE guidelines (1). Six hippocampal and six temporal lobe cortex paraffin embedded tissue samples from non-epilepsy control subjects (MAD: 61 years; SD: 6 F & 6 M, Table SI2) were obtained from The Netherlands Brain Bank (NBB), Netherlands Institute for Neuroscience, Amsterdam (open access: www.brainbank.nl). All material was collected from donors for whom a written informed consent for a brain autopsy and the use of the material and clinical information for research purposes had been obtained by the NBB.

IHC staining (DAB substrate, Thermon Fischer, Rockford, USA) was performed on tissue sections 4 mm in thickness (Leica RM2255 Mikrotom) and was evaluated using a tissue microarray including appendix, tonsil, liver, and pancreas on all slides with proteinatlas.org as reference (2). Clone names

and staining conditions are listed in Table SI4. The deparaffinization step was done using EZ Prep (Ventana, Arizona, USA), and staining was done on a Ventana BenchMark ULTRA (Ventana, Arizona, USA) visualized with Ventane Opitiview DAB IHC detection kit (Ventana, Arizona, USA). Epitope demasking was done by heat induced epitop retrievel using ULTRA CC1 buffer (Ventana, Arizona, USA). To differentiate between cortical layers and hippocampal regions all slides were counterstained with hematoxylin (3). Staining for marker expression (immunoreactivity) was determined semi quantitatively by absorbance measurement analysis on 10X digital microscopy images (pixel color intensity ranging from 0-255) using Zeiss ZenBlue software, where 0 represents maximal membranous and cytoplasmic staining and 255 represents no staining. Measurements were conducted as triplicates in neocortical layers 1, 2, 3, 5 & 6, and in hippocampal dentate gyrus molecular layer and hilus (Fig SI1;2). Measurements were normalized to the mean of triplicate measurements in white matter on each individual sample slides. In cases where slides did not include white matter, an average of white matter measurements from all slides was used alternatively for normalization. Unpaired multiple t-test with Welch correction (5% FDR) comparing expression of each marker in mTLE, non-epilepsy hippocampal dentate gyrus (hilus and molecular layer), and temporal lobe cortex (layers 1-3, 5 & 6) was performed using GraphPad Prism version 9.0.

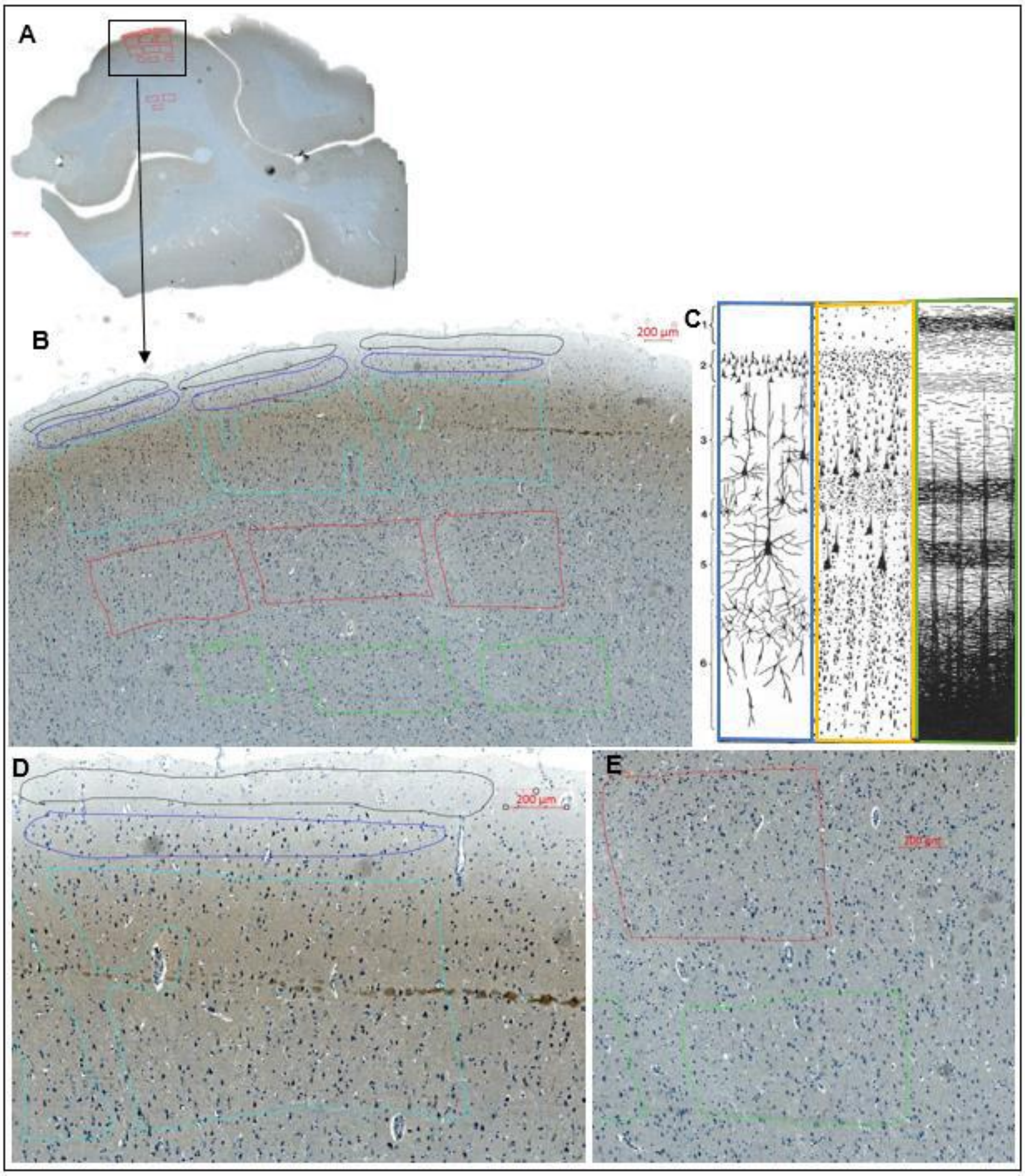

Fig SI1 Division of temporal lobe neocortical layers for absorbance measurements using a CACNB3 stained slice from a non-epilepsy subject as example. **A light field microscopy image of** an entire temporal lobe neocortical section. The red markings are measurements in resp. Layers 1,2,3,5,6, and white matter. **B** A section of Fig A where the layers can be seen up close. **C** A schematic drawing of a staining of the cells in the cortical layers. The numbers on the left indicate the cortical layers. The Fig is divided into three parts vertically. The blue square shows the distribution of neurons and axons in each layer. The yellow square only shows cell bodies. The green square shows myelin sheets. The Fig is from Moos el. al, Basal neuroanatomi. 4th edition. Kbh.: FADL; 2016 (4). **D** A section of Fig B with focus on layer 1,2 and 3**.** **E** A section of Fig B with focus on layer 5 and 6.

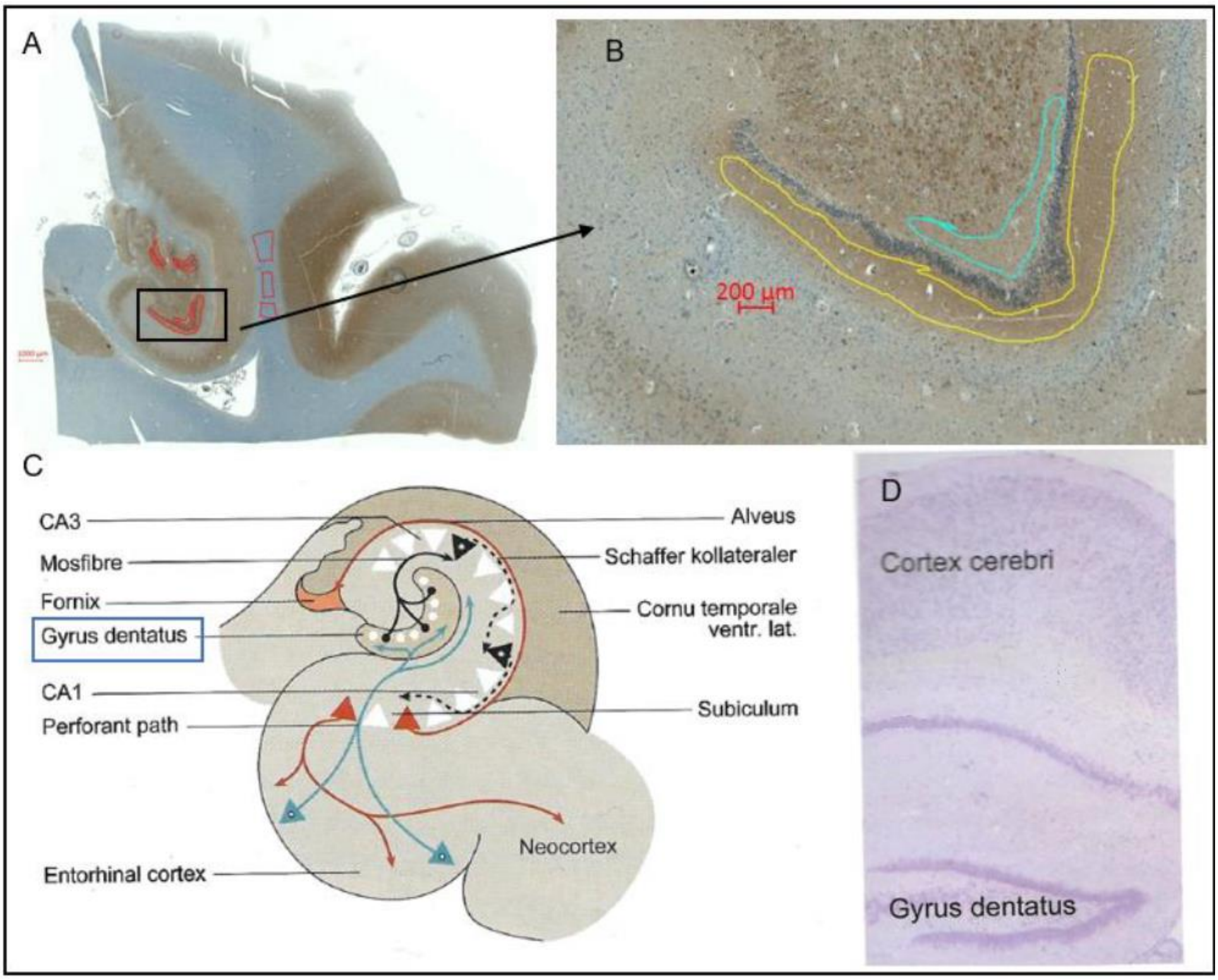

Fig SI2 The division of the hippocampus formation used for absorbance measurements using a CACNB3 stained slice from a non-epilepsy subject as example. **A light field microscopy image of an** entire hippocampal section. The red markings are measurements in hilus, molecular layer and white matter. **B** an up close section of Fig A with focus on gyrus dentatus. The light blue marking indicates a hilus measurement and the yellow marking indicates a molecular layer measurement. **C** A schematic drawing of the hippocampal formation. D. an immunohostochemical Toluidin blue staining of a hippocampal slice. Fig C og D (slightly modified) is from Moos el. al, Basal neuroanatomi. 4th edition. Kbh.: FADL; 2016 (4).

Method S3

Western blots

Protein was extracted from dissected hippocampal and temporal lobe neocortical paired tissue samples from 17 mTLE (MAS: 42 years; SD: 10 F & 7 M, Table 1) and 16 non-epilepsy control subjects (MAD: 39 years; SD: 7 F & 9 M, Table SI2) by the AllPrep DNA/RNA/Protein Extraction Kit (Qiagen) according to the manufacturer's instructions. The buffer was subsequently changed to

SDS (5%) using Centri-Sep Spin columns (Thermo Fisher, ref: 401762). The protein content in each supernatant fraction was determined using a BCA protein assay kit (Thermo Fisher, ref: 23235), and samples were loaded at 20 µg protein per well. Gel electrophoreses, transfer, blocking, incubation with antibodies and visualization were performed as previously described (5). Primary antibodies were CACNB3 anti-rabbit (Invitrogen; PA5-77301) and Vinculin anti-mouse (Sigma; V9131). Secondary antibodies were polyclonal secondary horseradish peroxidase (HRP)-conjugated goat anti-mouse antibody (P0447) and pig anti-rabbit antibody (P0217) (both Agilent, former Dako; Glostrup, Denmark).

Fig SI3-16 Principal component analysis (PCA) plots

PCA plots of RNA-Seq read counts from 17 mTLE cortical samples (C1, C2, C3…), 17 mTLE hippocampal samples (H1, H2, H3…) and two brain cancer cortical samples (CTRL1 & CTRL2). The PCA plots illustrate the following clinical parameters: patients age at operation, age at seizure debut, etiology, most frequent seizure type, seizure frequency per month, use of tobacco, use of alcohol, drug abuse, hippocampal sclerosis diagnosis, learning disabilities, impaired language abilities, impaired memory, and psychiatric co-morbidities.

Expected gene expression differences related to both tissue type and gender (we compared different anatomical areas and X & Y chromosomes express different genes, respectively) were the strongest drivers in the Kjær *et al.* dataset, while no other clinical parameters investigated influenced the result of the Kjær *et al.* mTLE transcriptome dataset in a significant manner (Fig SI3-16).

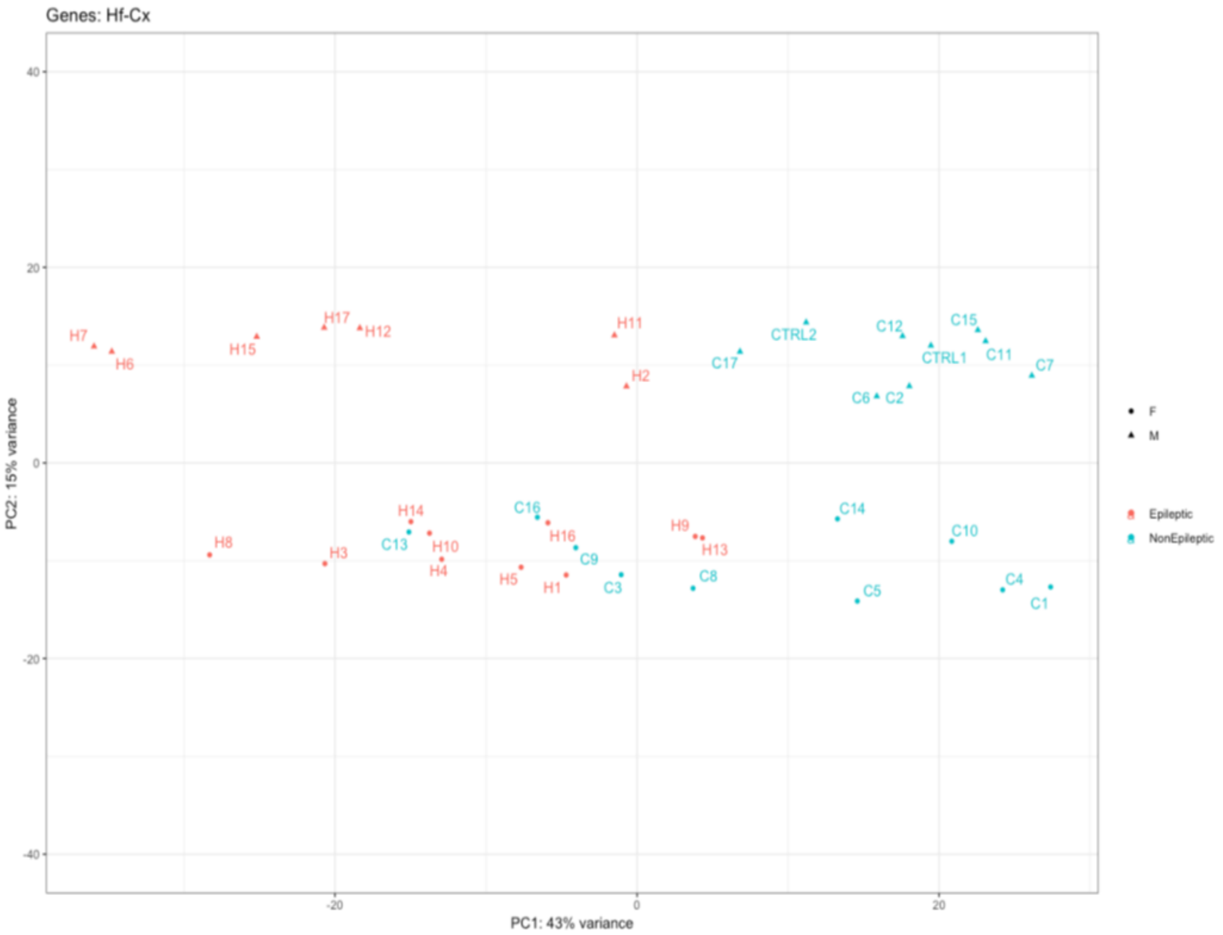

Fig SI3 PCA of mTLE patient sex and tissue. The result is based on transcriptome read counts of individual genes among 17 mTLE cortical samples (C1, C2, C3…), 17 mTLE hippocampal samples (H1, H2, H3…) and two brain cancer cortical samples (CTRL1 & CTRL2). S, sex; Circles, female (F);

Triangles, male (M); Red, hippocampal tissue (Epileptic); Turquoise, cortical tissue (NonEpileptic); The PCA shows clusters of samples based on their similarity. PC1, the line in the K-dimensional variable space that best approximates the data in the least squares sense, which in this Fig represent 43% of the variation found in the data set; PC2, a line in the K-dimensional variable space, which is orthogonal to the first PC represent 15% of the variation found in the data set.

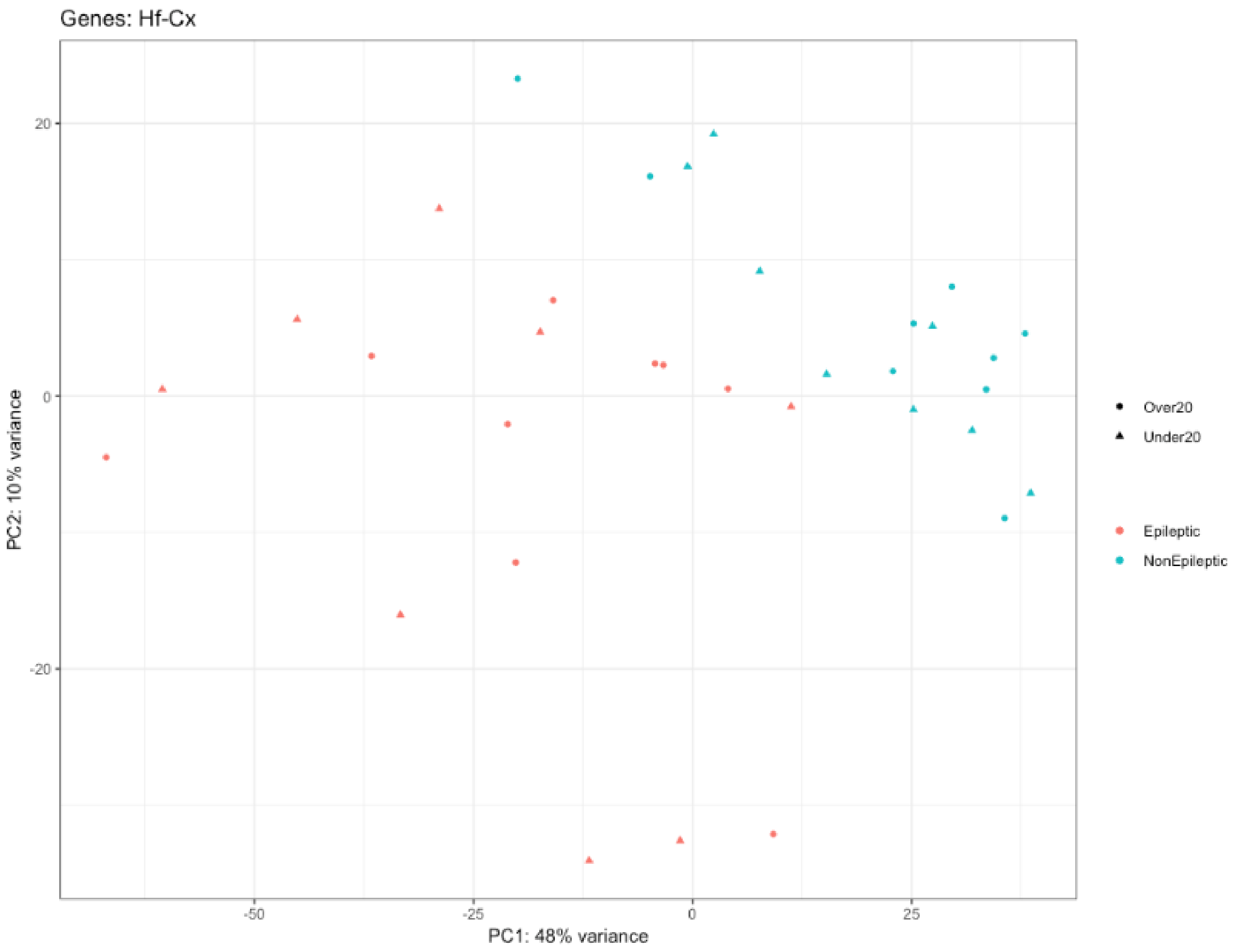

Fig SI4. PCA of mTLE patients age at seizure debut. Circles, patients that are > 20 years of age; Triangles, patients < 20 years of age. Red, hippocampal tissue (Epileptic); Turquoise, cortical tissue (NonEpileptic); The PCA shows clusters of samples based on their similarity. PC1 represent 43% of the variation found in the data set. PC2 represent 10% of the variation found in the data set.

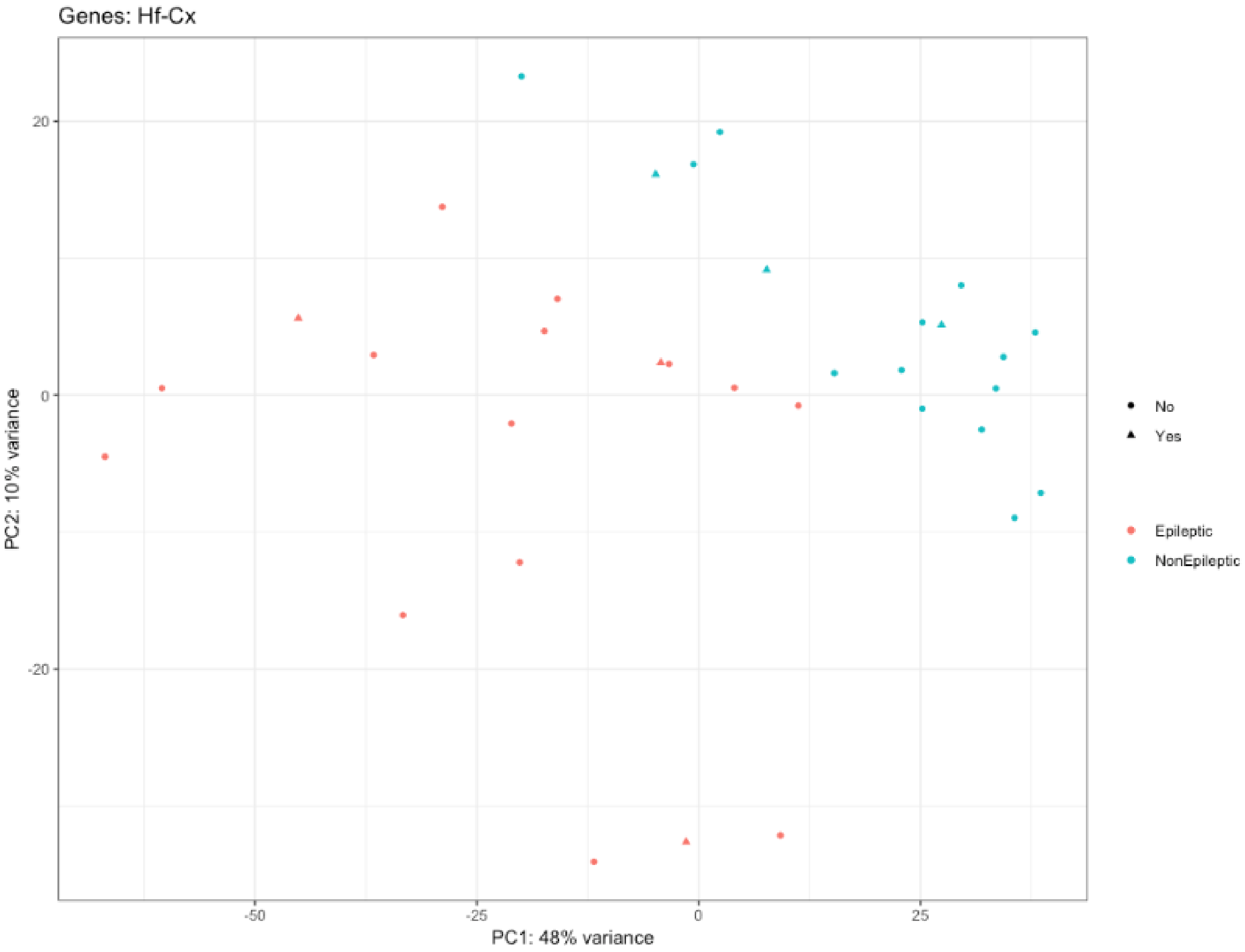

Fig SI5. PCA of mTLE patient etiology. Circles, No (unknown etiology); Triangles, Yes (known etiology). Red, hippocampal tissue (Epileptic); Turquoise, cortical tissue (NonEpileptic). The PCA shows clusters of samples based on their similarity. PC1 represent 43% of the variation found in the data set. PC2 represent 10% of the variation found in the data set.

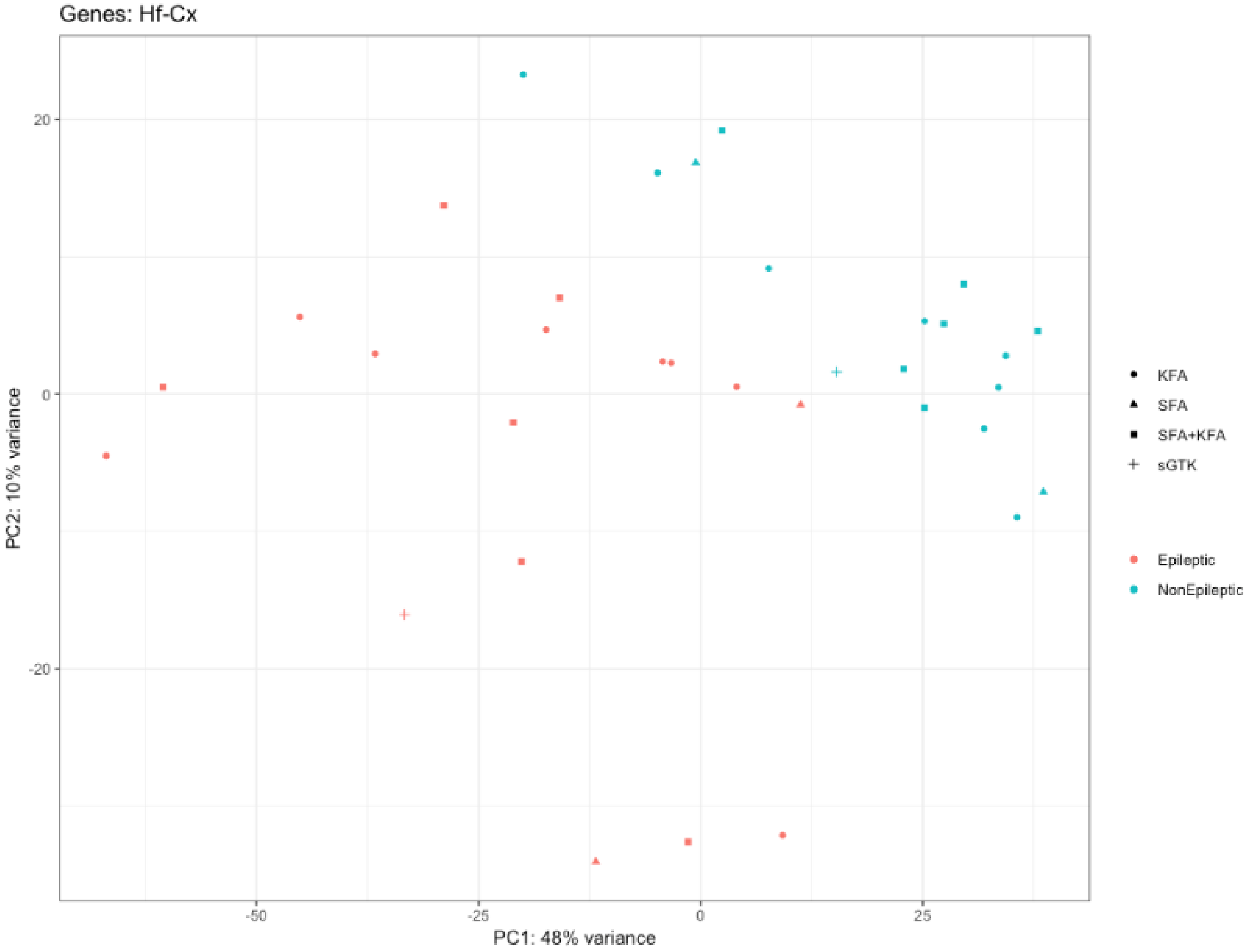

Fig SI6. PCA of mTLE patients most frequent seizure type. Circles, KFA (FoA: focal onset aware (seizure classification (1)); triangles, SFA (FoU: focal onset impaired awareness (seizure classification (1)); Squares, SFA+KFA (FoA +FoU); plusses, sGTK (FtBTC: focal to bilateral tonic clonic (seizure classification (1)); red, hippocampal tissue (Epileptic); turquoise, cortical tissue (NonEpileptic). The PCA shows clusters of samples based on their similarity. PC1 represent 43% of the variation found in the data set. PC2 represent 10% of the variation found in the data set.

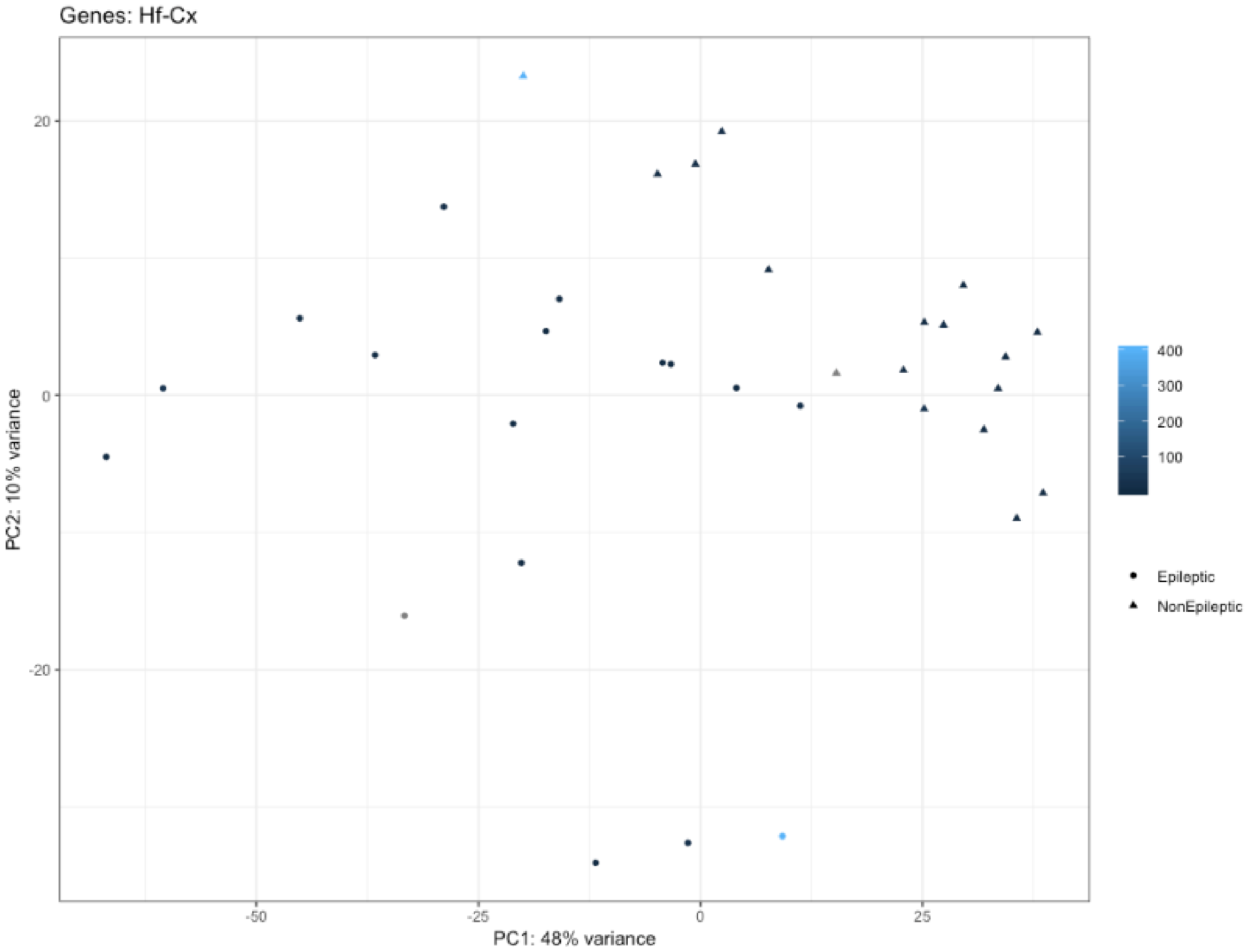

Fig SI7. PCA of mTLE patient seizure frequency per month. Scalebar to the right shown seizure frequencies from 0-400 seizures per month: the darker the blue color the fewer seizures and the lighter the blue color the more seizures. Circles, hippocampal tissue (Epileptic); triangles, cortical tissue (NonEpileptic); The PCA shows clusters of samples based on their similarity. PC1 represent 43% of the variation found in the data set. PC2 represent 10% of the variation found in the data set.

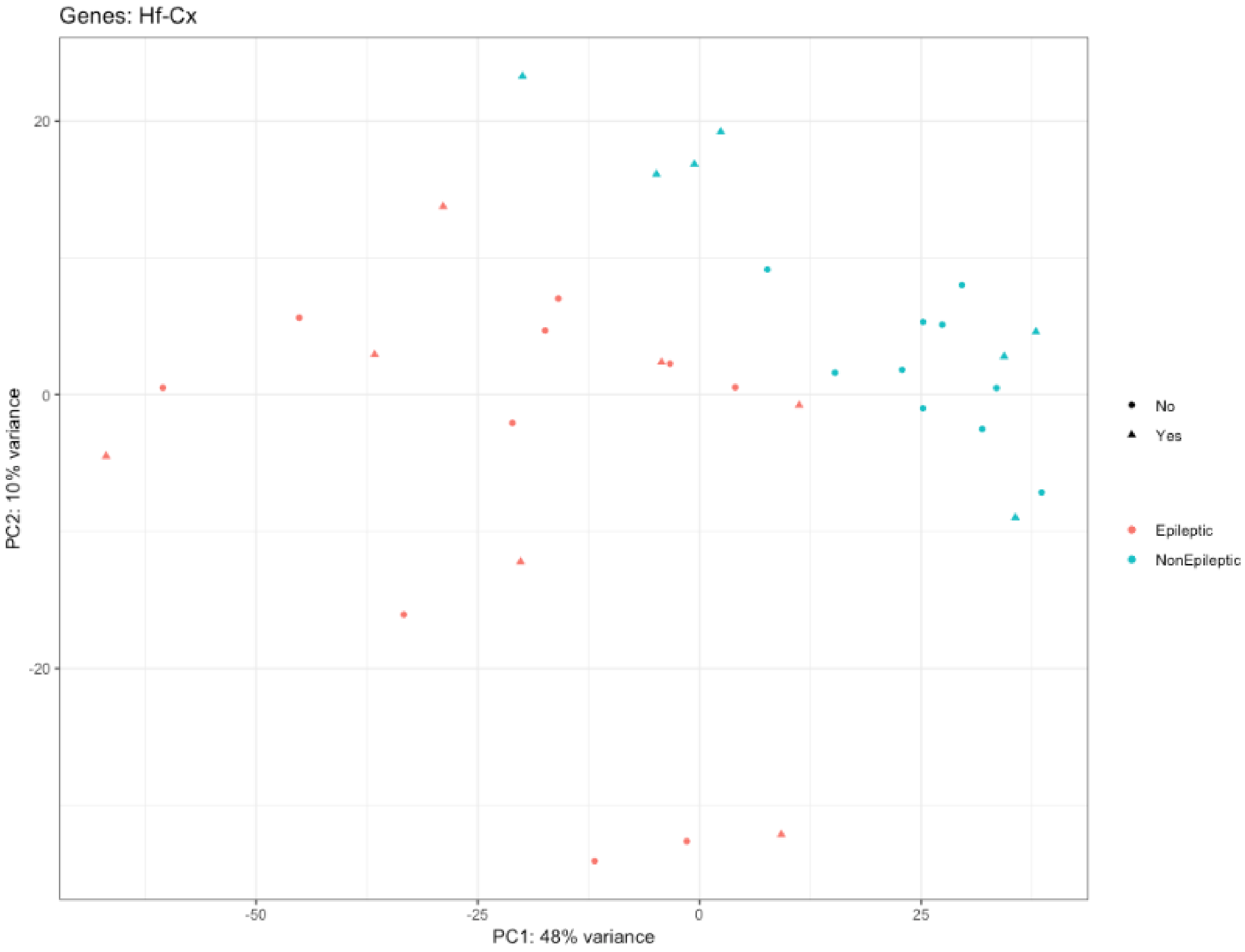

Fig SI8. PCA of mTLE patients use of tobacco. Circles, No (no use of tobacco); triangles,Yes (use tobacco); red, hippocampal tissue (Epileptic); turquoise, cortical tissue (NonEpileptic); The PCA shows clusters of samples based on their similarity. PC1 represent 43% of the variation found in the data set. PC2 represent 10% of the variation found in the data set.

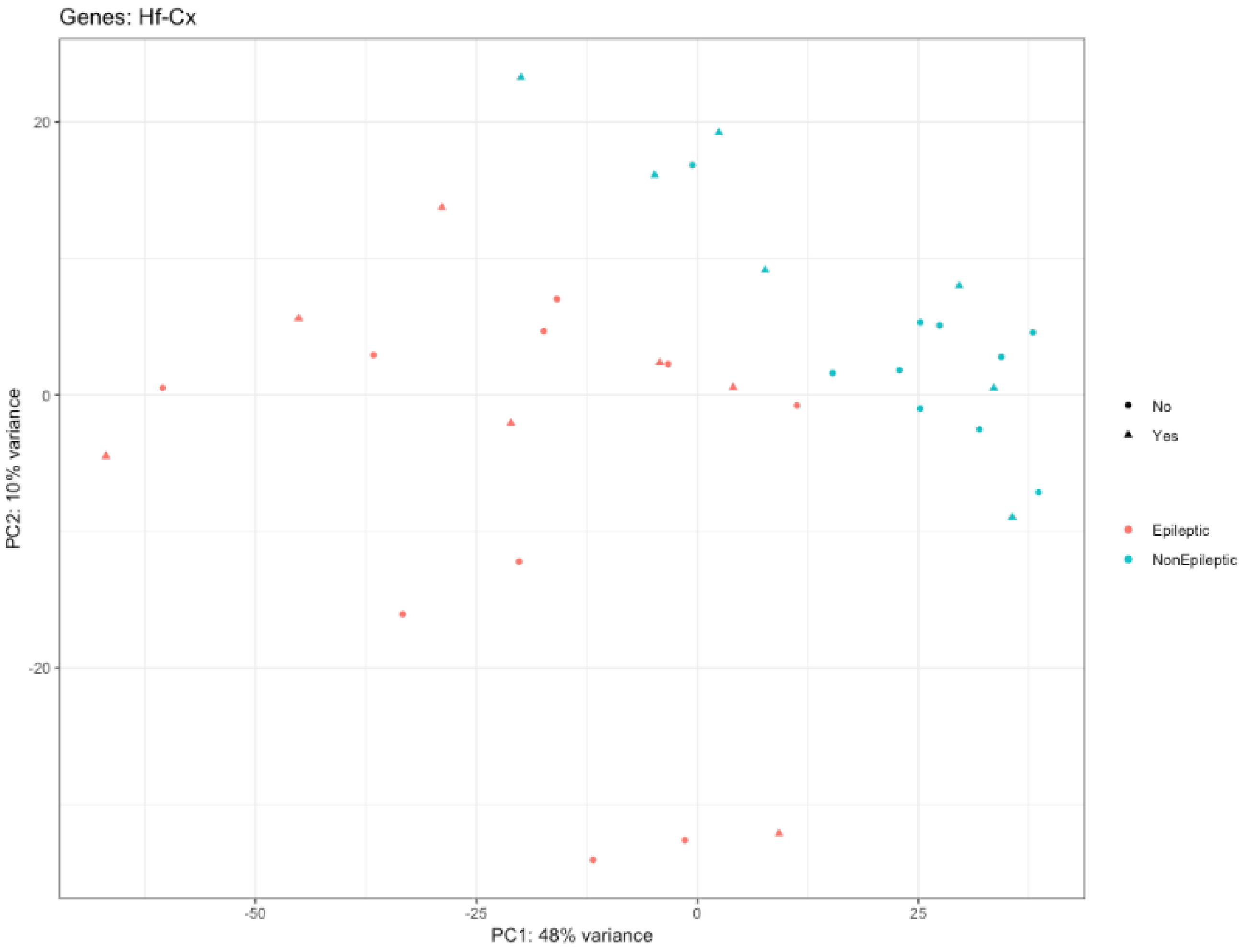

Fig SI9. PCA of mTLE patients use of alcohol. Circles, No (no use of alcohol); triangles, Yes (use alcohol); red, hippocampal tissue (Epileptic); turquoise, cortical tissue (NonEpileptic); The PCA shows clusters of samples based on their similarity. PC1 represent 43% of the variation found in the data set. PC2 represent 10% of the variation found in the data set.

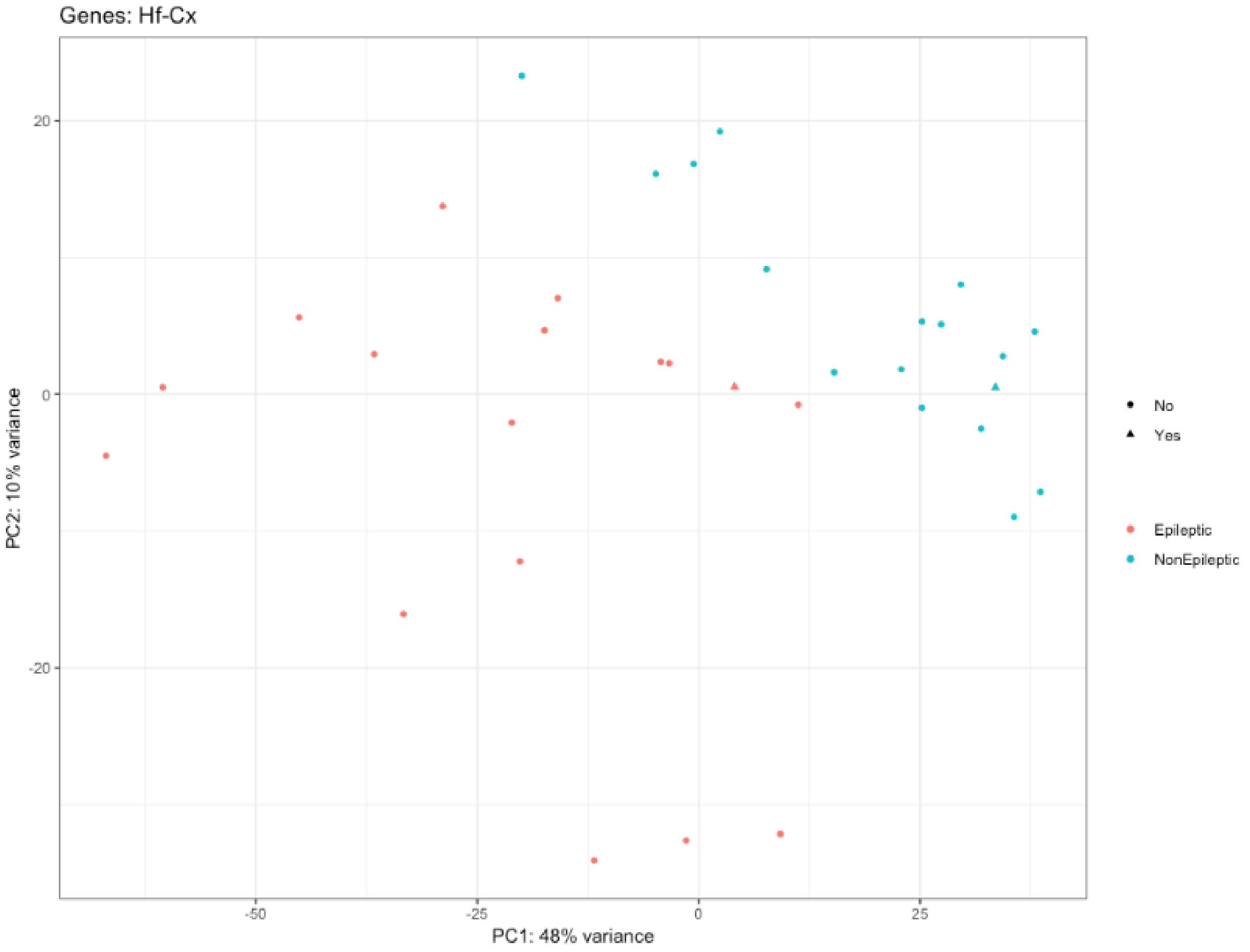

Fig SI10. PCA of mTLE patients drug abuse. Circles, No (no drug abuse); triangle, Yes (drug abuse); red, hippocampal tissue (Epileptic); turquoise, cortical tissue (NonEpileptic). The PCA shows clusters of samples based on their similarity. PC1 represent 43% of the variation found in the data set. PC2 represent 10% of the variation found in the data set.

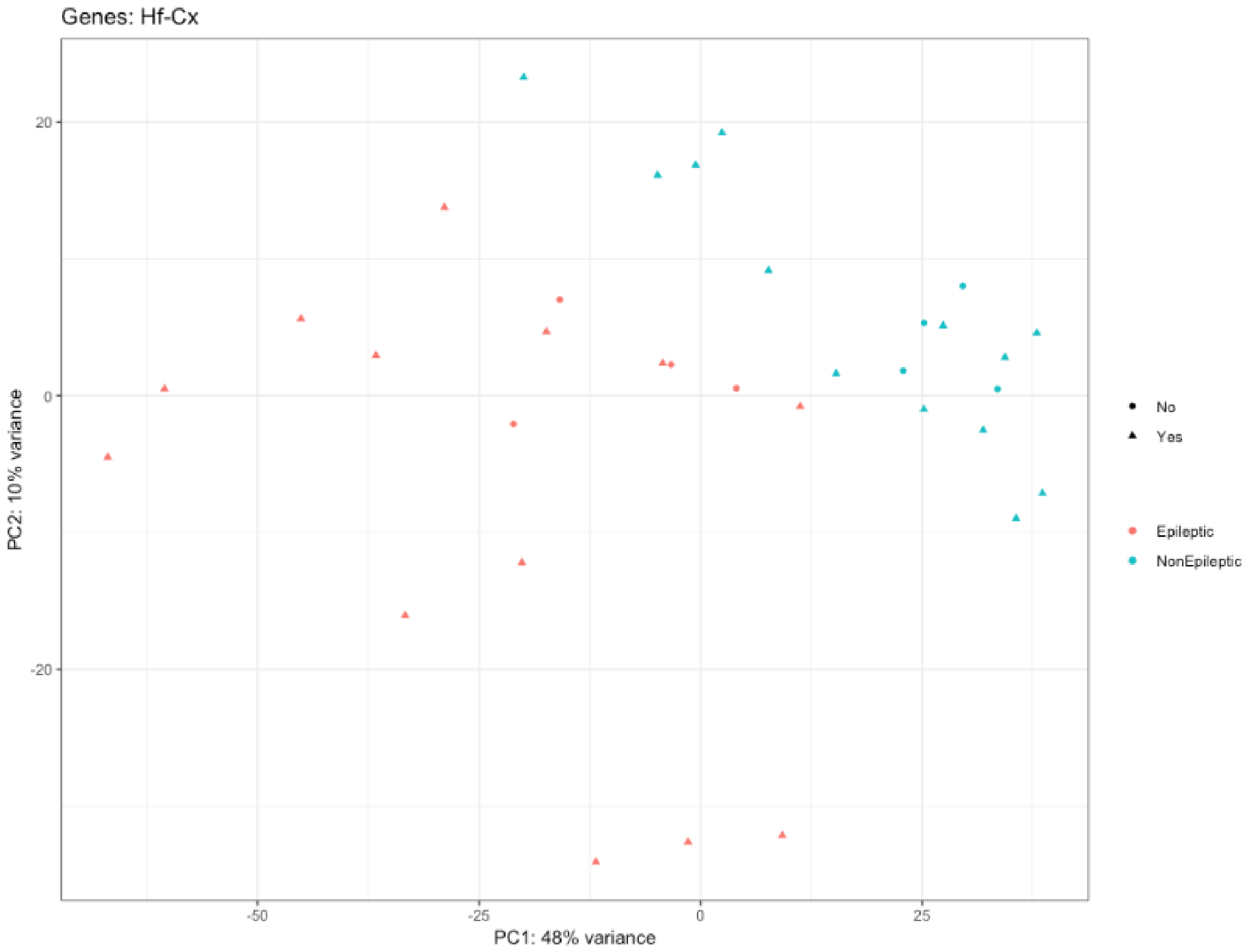

Fig SI11. PCA of mTLE patients diagnosed with or without hippocampal sclerosis (HS). Circles, No (no HS diagnosis); triangle, Yes (HS diagnosis); red, hippocampal tissue (Epileptic); turquoise, cortical tissue (NonEpileptic). The PCA shows clusters of samples based on their similarity. PC1 represent 43% of the variation found in the data set. PC2 represent 10% of the variation found in the data set.

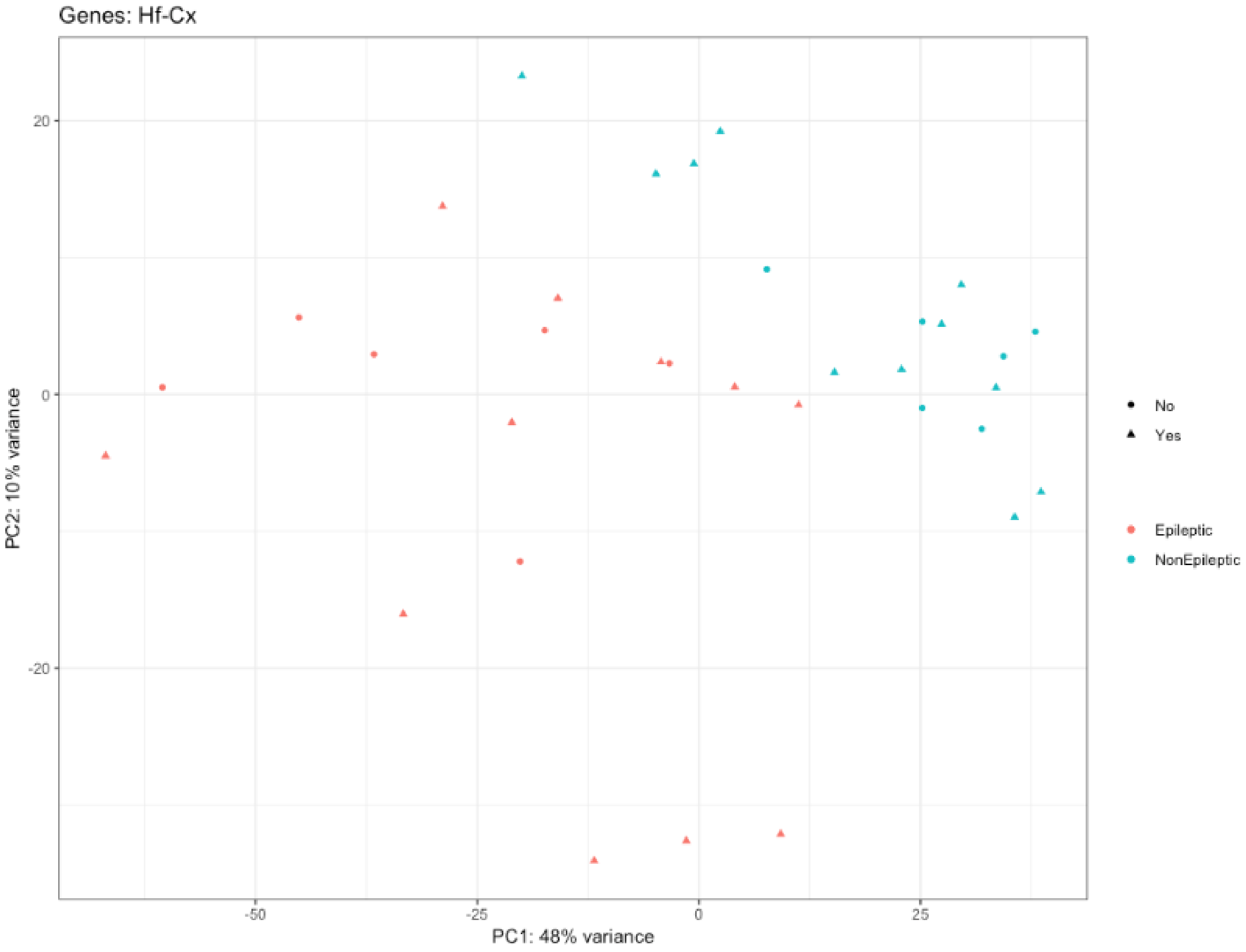

Fig SI12. PCA of mTLE patients diagnosed with impaired language ability (ILA) diagnosis. Circles, No (no ILA diagnosis); triangle, Yes (ILA diagnosis); red, hippocampal tissue (Epileptic); turquoise, cortical tissue (NonEpileptic). The PCA shows clusters of samples based on their similarity. PC1 represent 43% of the variation found in the data set. PC2 represent 10% of the variation found in the data set.

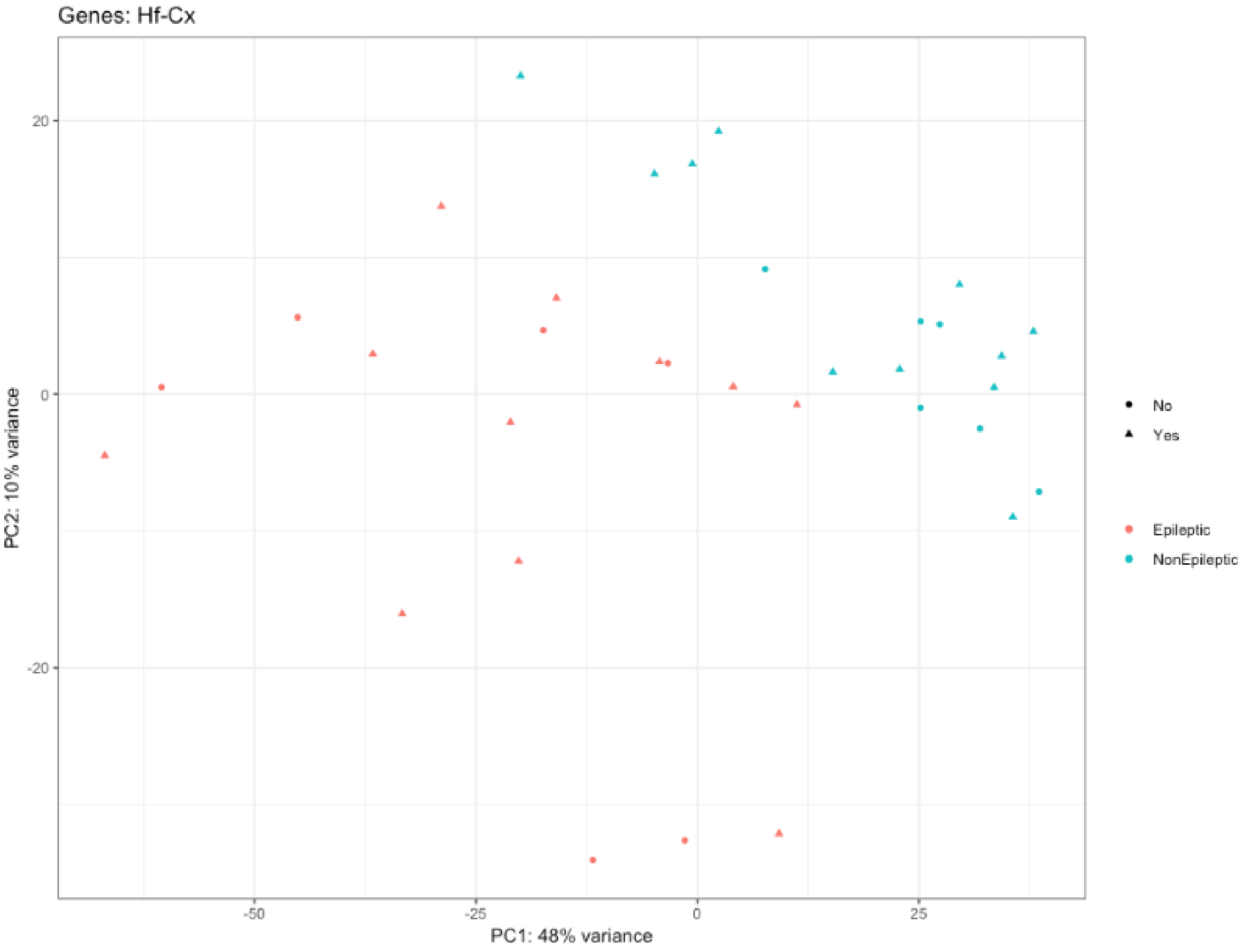

Fig SI13. PCA of mTLE patients diagnosed with impaired memory (IM) diagnosis. Circles, No (no IM diagnosis); triangle, Yes (IM diagnosis). Red, hippocampal tissue (Epileptic); turquoise, cortical tissue (NonEpileptic). The PCA shows clusters of samples based on their similarity. PC1 represent 43% of the variation found in the data set. PC2 represent 10% of the variation found in the data set.

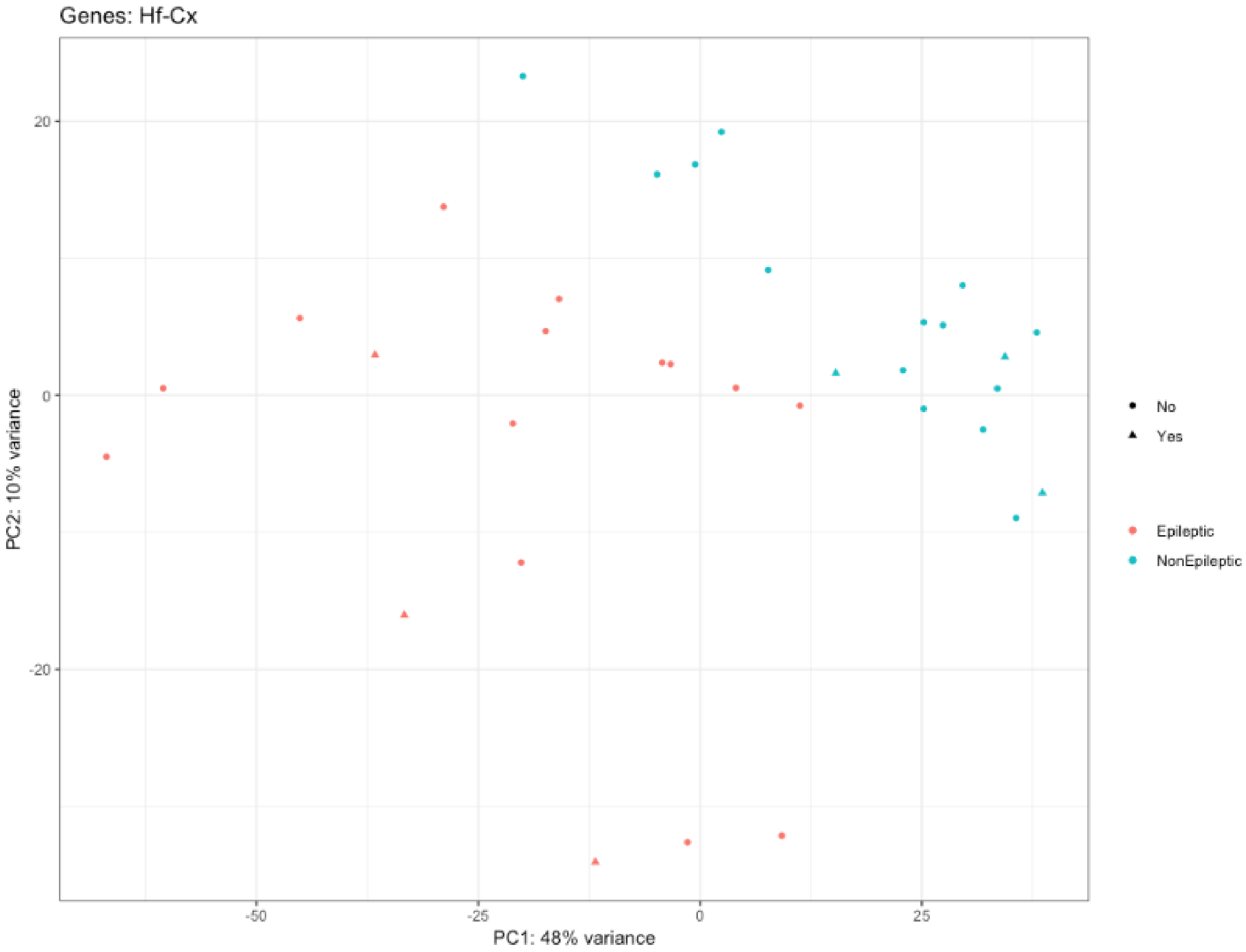

Fig SI14. PCA of mTLE patients diagnosed with learning disabilities (LD) diagnosis. Circles, No (no LD diagnosis); triangle, Yes (LD diagnosis); red, hippocampal tissue (Epileptic); turquoise: cortical tissue (NonEpileptic). The PCA shows clusters of samples based on their similarity. PC1 represent 43% of the variation found in the data set. PC2 represent 10% of the variation found in the data set.

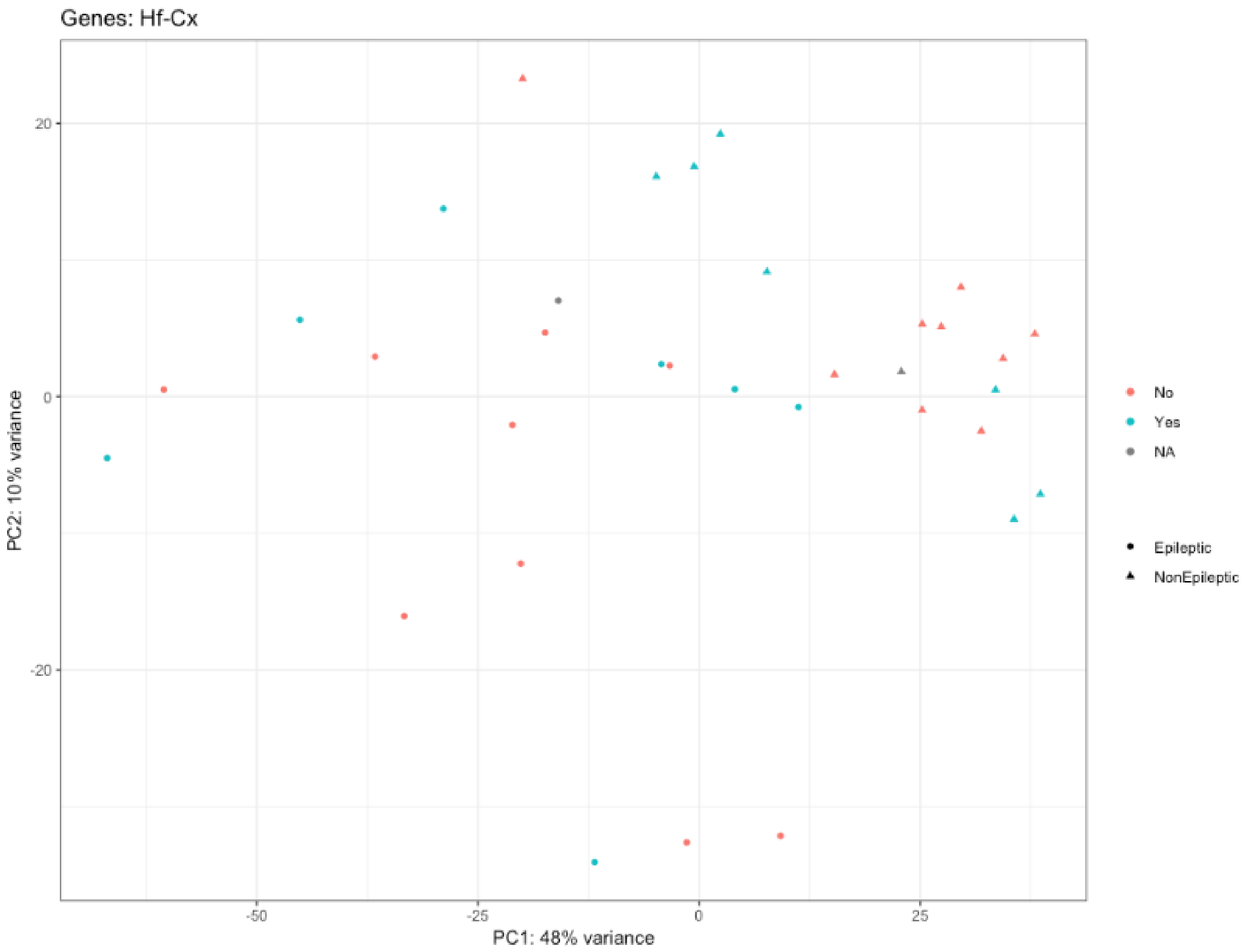

Fig SI15. PCA of mTLE patients diagnosed with psychiatric co-morbidity diagnosis such as attention deficit hyperactivity disorder (ADHD), anxiety, depression, psychosis, or post-traumatic stress syndrome (PTSD). Red and grey circles and triangles, No and NA (no diagnosed psychiatric co-morbidity); turquoise circles and triangles, Yes (diagnosed with a psychiatric co-morbidity); circles, hippocampal tissue (Epileptic); triangles, cortical tissue (NonEpileptic). The PCA shows clusters of samples based on their similarity. PC1 represent 43% of the variation found in the data set. PC2 represent 10% of the variation found in the data set.

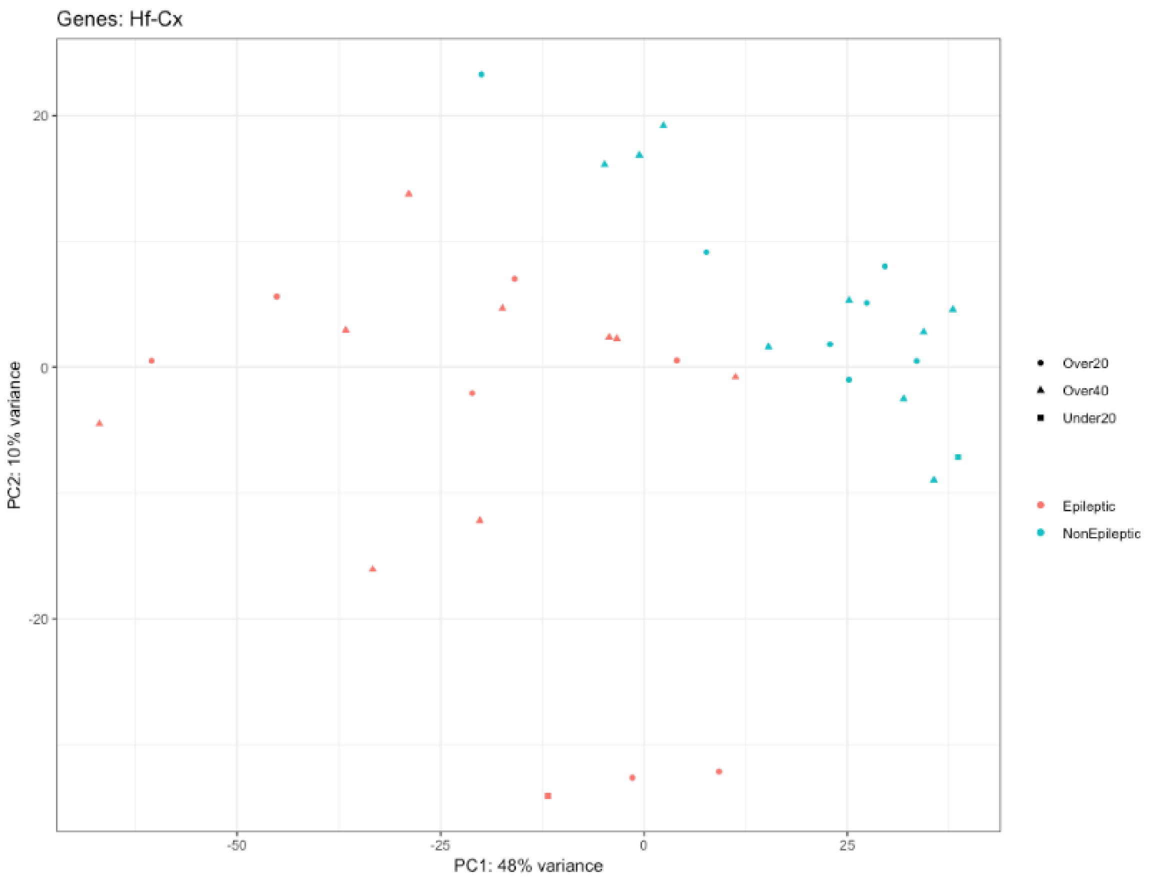

Fig SI16. PCA of mTLE patients age at operation. Circles, > 20 years of age; triangles, > 40 years of age; square, < 20 years of age; red, hippocampal tissue (Epileptic); turquoise, cortical tissue (NonEpileptic). The PCA shows clusters of samples based on their similarity. PC1 represent 43% of the variation found in the data set. PC2 represent 10% of the variation found in the data set.

Selection of DEGs by benchmarking

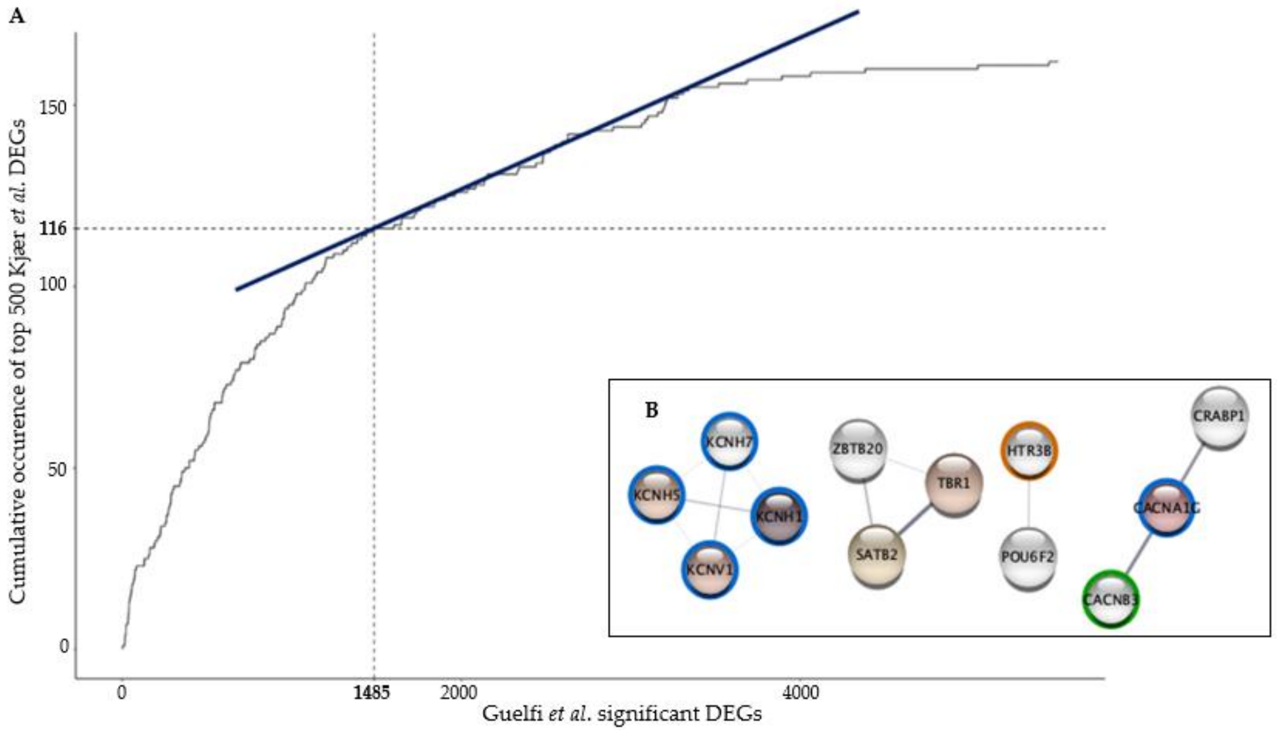

Fig SI17. Lead target identification. A. **Benchmark plot**. The plot shows the cumulative occurrence of the Kjær *et al*. top 500 significant DEGs* ranked by distance from origin** over the full list of DEGs reported by Guelfi *et al.*, sorted by absolute Log2FC. The intersection of the vertically and horizontally dashed lines indicates the first position along Guelfi *et al.*'s ranking where the frequency of Kjær *et al*. genes drop notably. *Significance defined by the S curve seen on the volcano plot (**Fig SI18**); ** this is the distance from the origin of the axes in the volcano plot (**Fig SI18**). **B**. **STRING modules comprising the mTLE lead targets: CACNB3, KCNH5, KCNH7, HTR3B and ZBTB20.** Network nodes represent genes. Edges represent protein-protein interactions. Proteins jointly contribute to shared function but do not necessarily bind each other physically. Saturated node color: very well described in epilepsy; Pale node color: rarely described in relation to epilepsy; No node color: no known connection to epilepsy; Blue encircled nodes, FDA approved drugs target the gene product; orange encircled nodes, a chemical compound modulate the gene product; green encircled nodes, the gene product was biologically characterized.

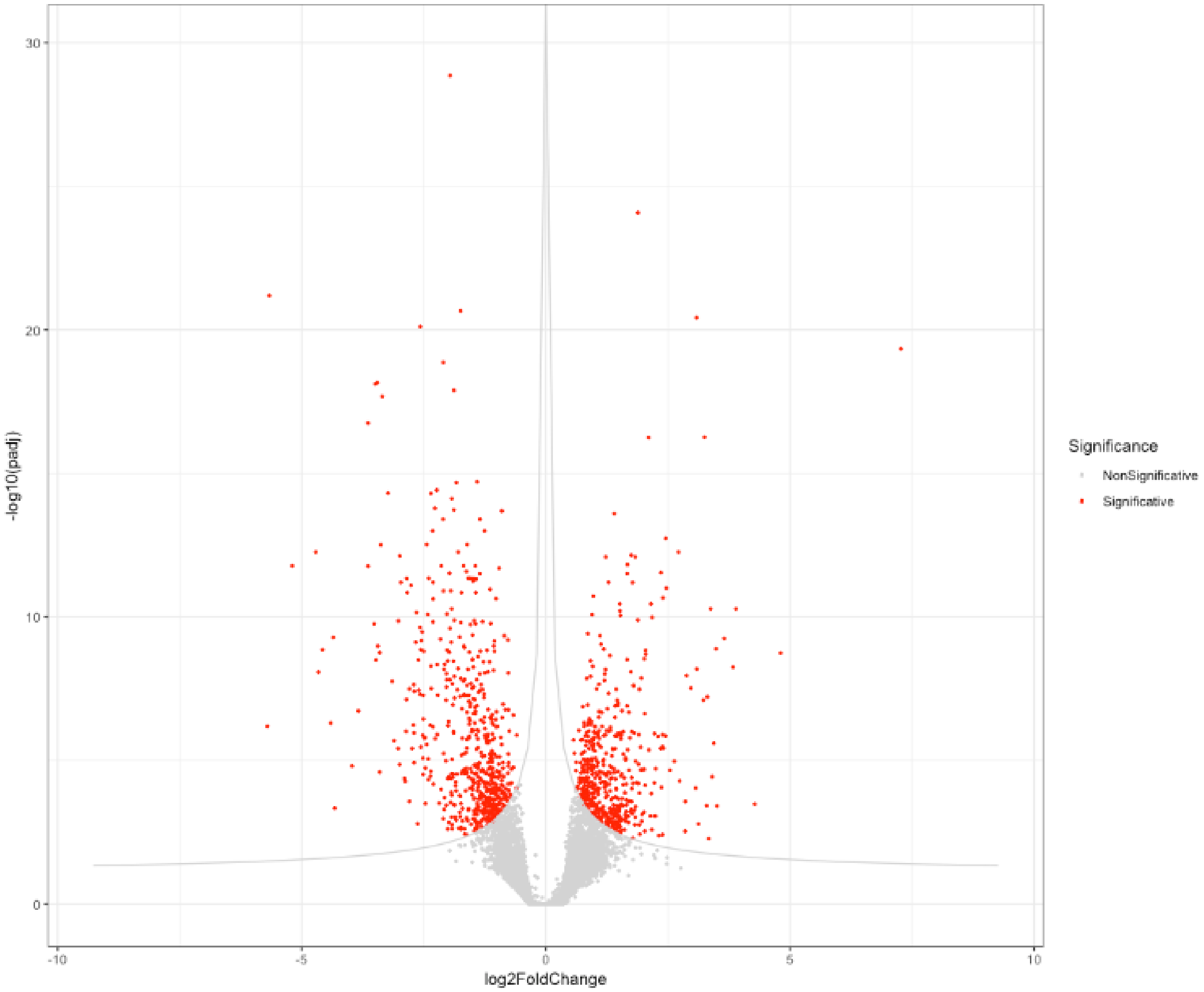

Fig SI18. Volcano plot. The plot show transcriptome data from 17 mTLE patients (2) with 0,05 p-values threshold and no LFC setting. In red is highlighted the significant genes defined as the ones lying above the line defined by the following equation: $-\log10(a) + 1/(b^*(|x|^c))$, where *a* is the p-value threshold (0.05), *b* is a parameter setting the distance from y = 0 and *c* defines the curvature. Y-axis display magnitude of DEGs ($\log_2$(fold change) and x-axis display significance ($-\log_{10}$(p-value).

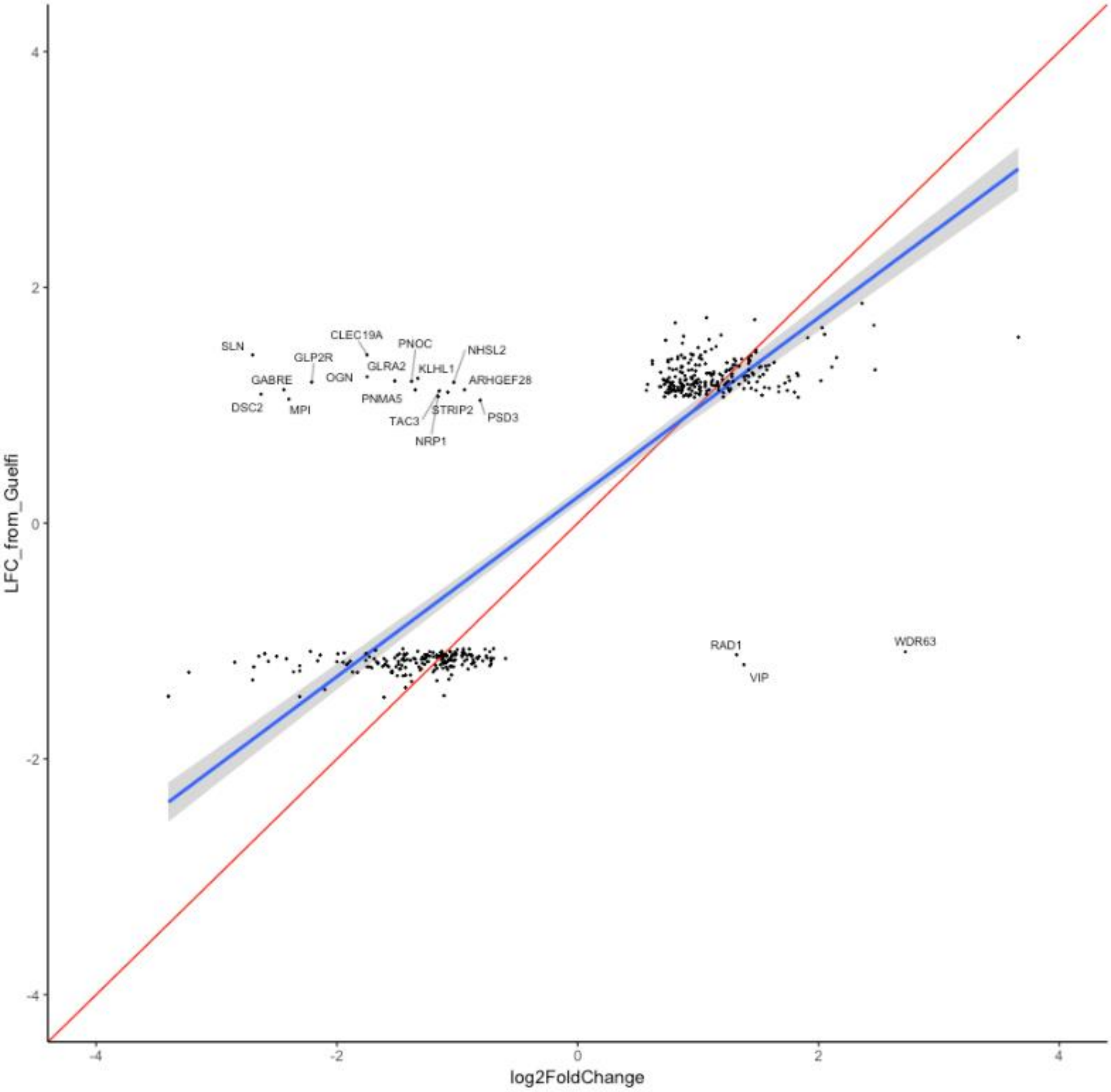

Fig SI19. Consensus plot. The plot shows the validated DEGs using the cut-off list from Fig SI17 (1,485 DEGs), where the Log2FC associated to them, according to the Kjær *et al*. study, was compared to the Log2FC found by Guelfi *et al.* Red line, perfect agreement (identity line x = y); Black dots, represent genes which are placed on the red line if the data sets agree not just on direction but also on value; The blue line, the actual trend; The grey surrounding, uncertainties taken into account; Annotated genes, represent DEGs with inconsistency in direction of regulation between data sets.

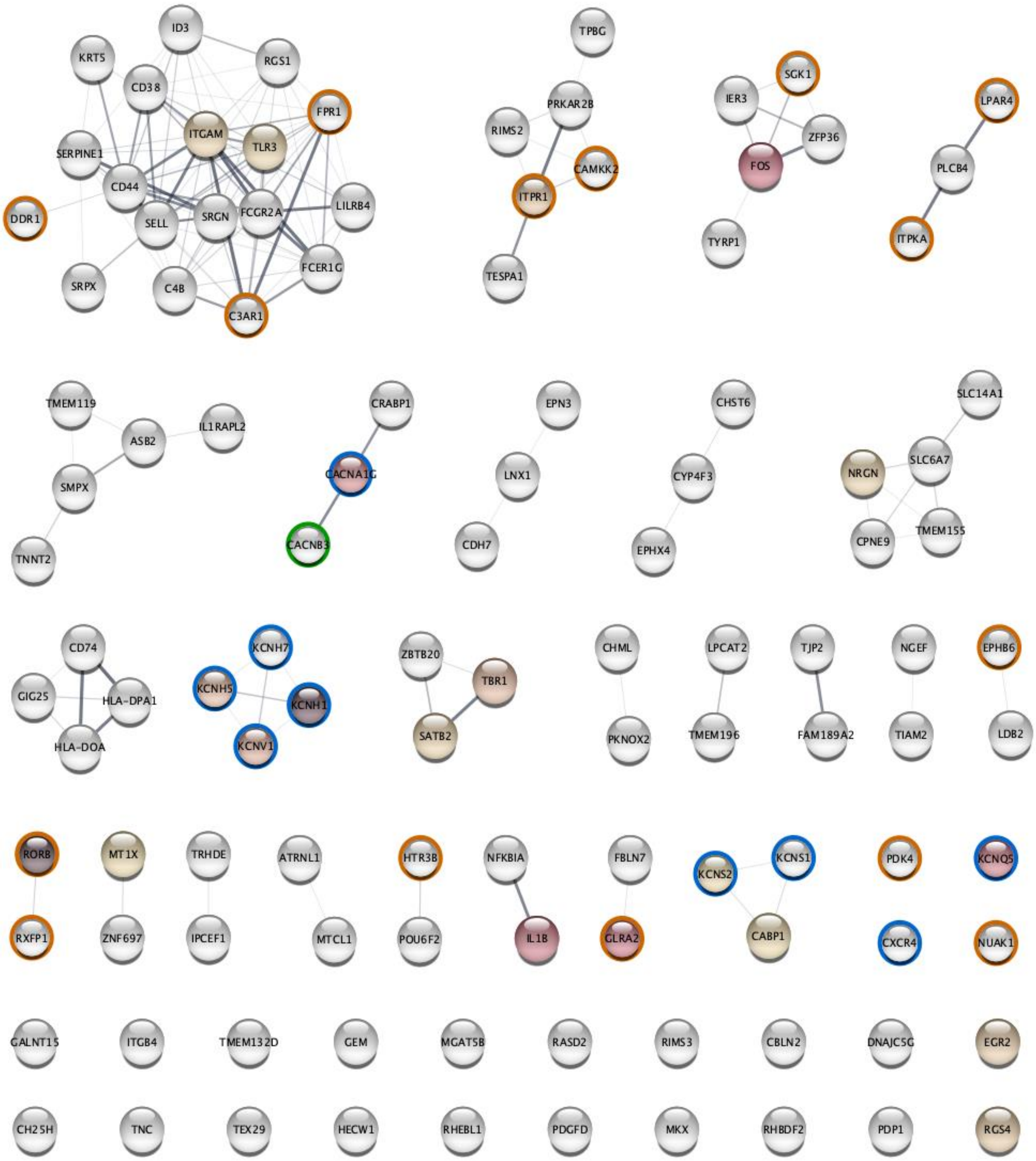

Fig SI20. Illustration of STRING network comprising 113 mTLE DEGs identified by consensus. Network nodes represent genes. Edges represent protein-protein interaction. Proteins jointly contribute to shared function but does not necessarily bind each other physically. Saturated node color means very well described in epilepsy, pale node color means rarely described in relation to epilepsy, and no node color means no known connection to epilepsy. Blue encircled nodes, FDA approved drugs target the gene product; orange encircled nodes, a chemical compound modulate the gene product; green encircled nodes, the gene product is biologically characterized.

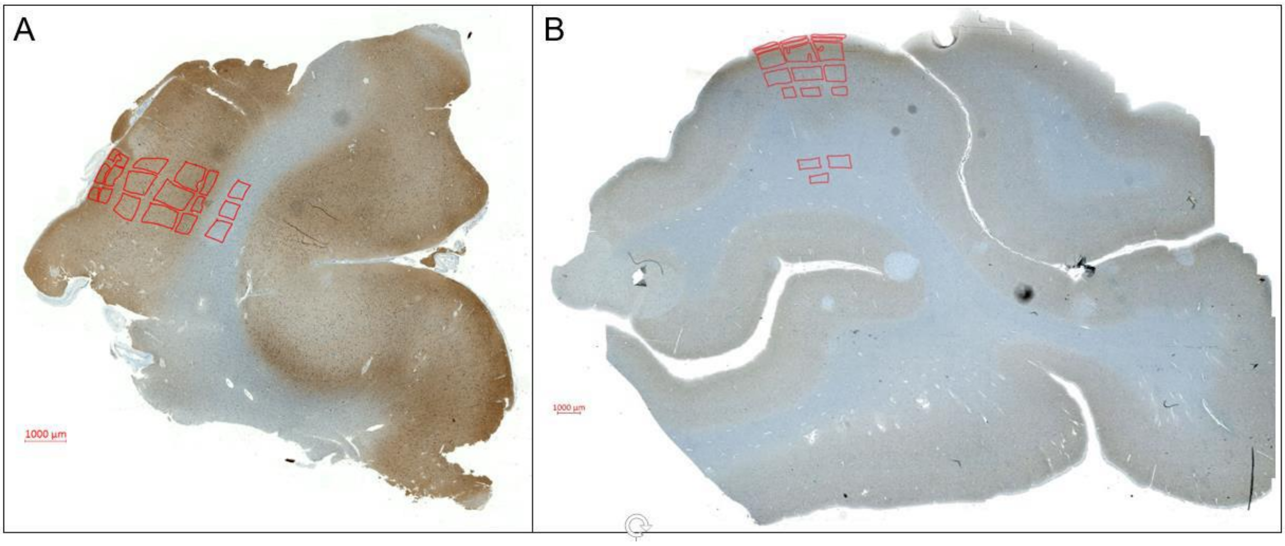

Fig SI21. Examples of light field microscopy images of temporal lobe neocortical IHC stained sections for CACNB3. A section from a mTLE patient. **B** section from a non-epilepsy subject. The red markings are measurements in resp. Layers 1,2,3,5,6, and white matter**.**

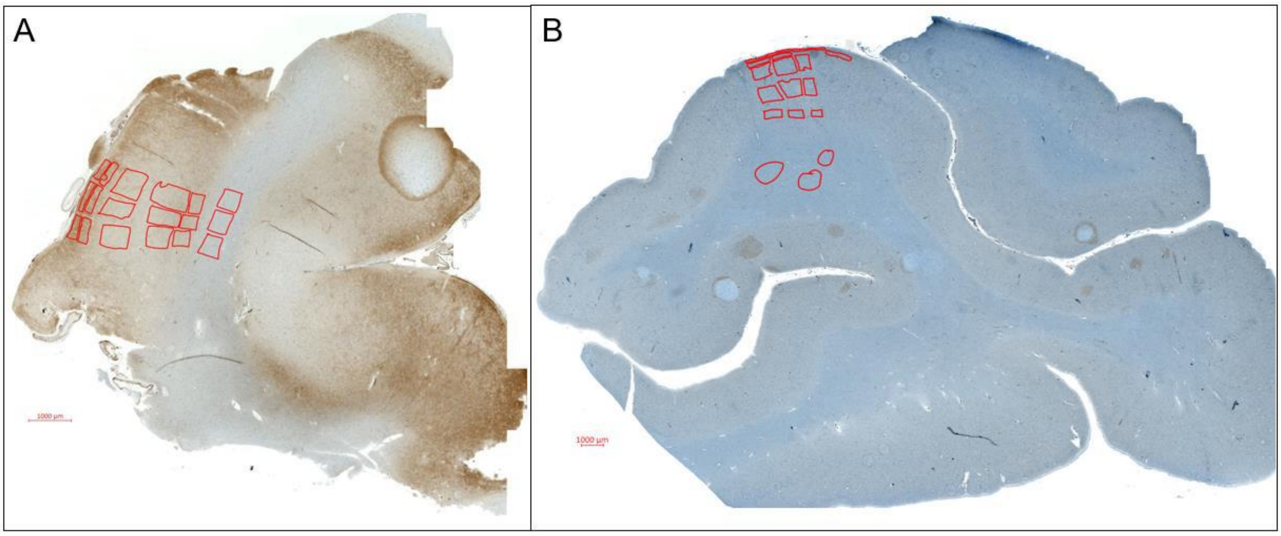

Fig SI22. Examples of light field microscopy images of temporal lobe neocortical IHC stained sections for KCNH5. **A** section from a mTLE patient. **B** section from a non-epilepsy subject. The red markings are measurements in resp. Layers 1,2,3,5,6, and white matter**.**

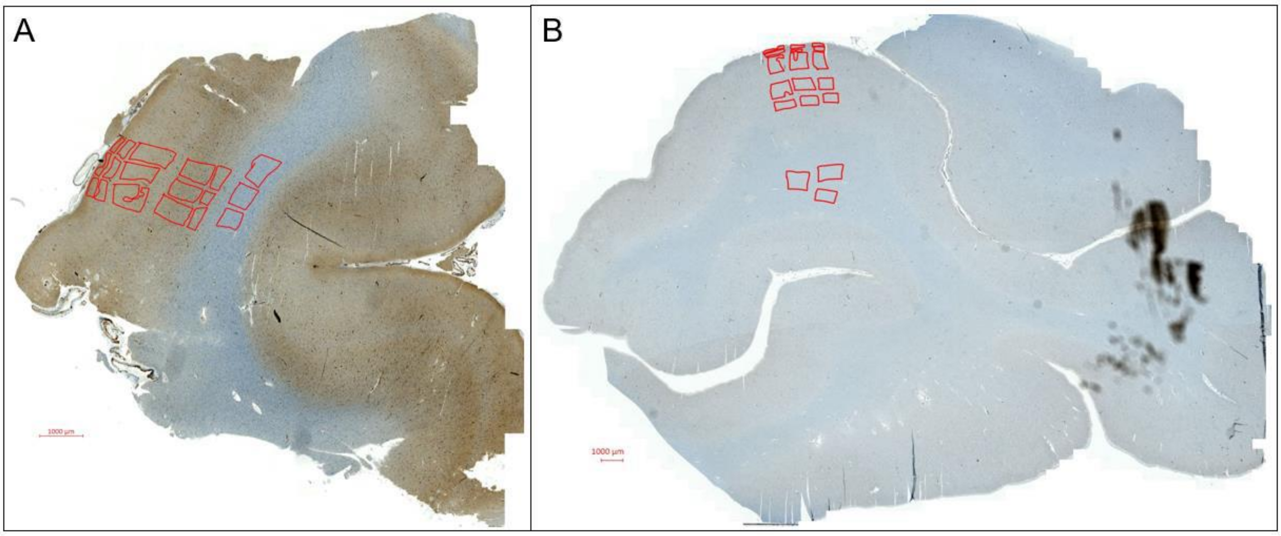

Fig SI23. Examples of light field microscopy images of temporal lobe neocortical IHC stained sections for KCNH7. **A** section from a mTLE patient. **B** section from a non-epilepsy subject. The red markings are measurements in resp. Layers 1,2,3,5,6, and white matter**.**

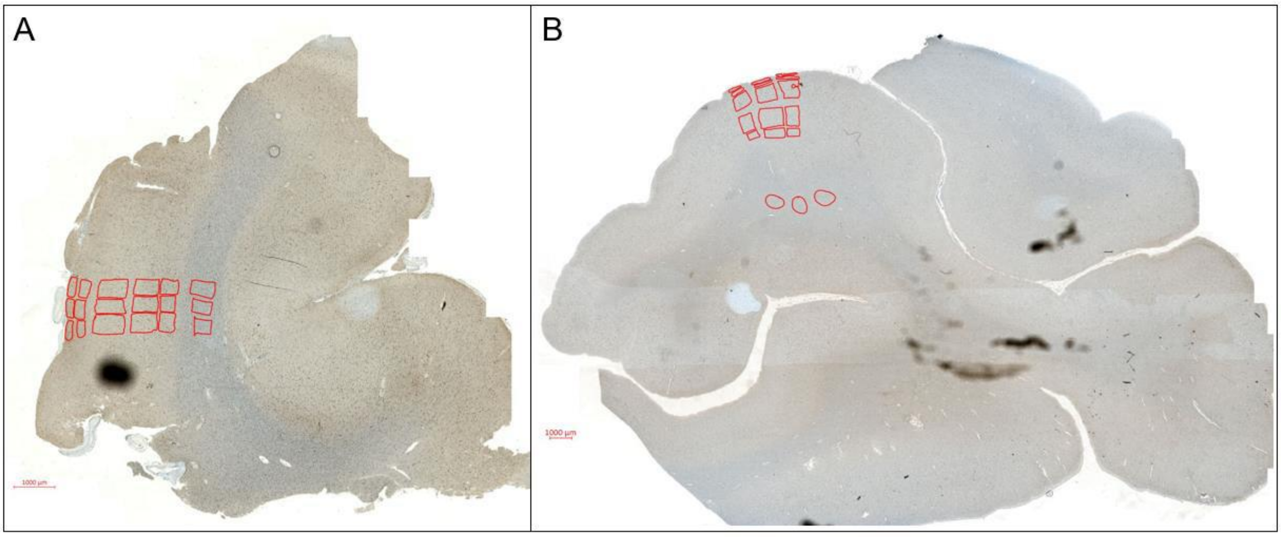

Fig SI24. Examples of light field microscopy images of temporal lobe neocortical IHC stained sections for HTR3B. A section from a mTLE patient. **B** section from a non-epilepsy subject. The red markings are measurements in resp. Layers 1,2,3,5,6, and white matter**.**

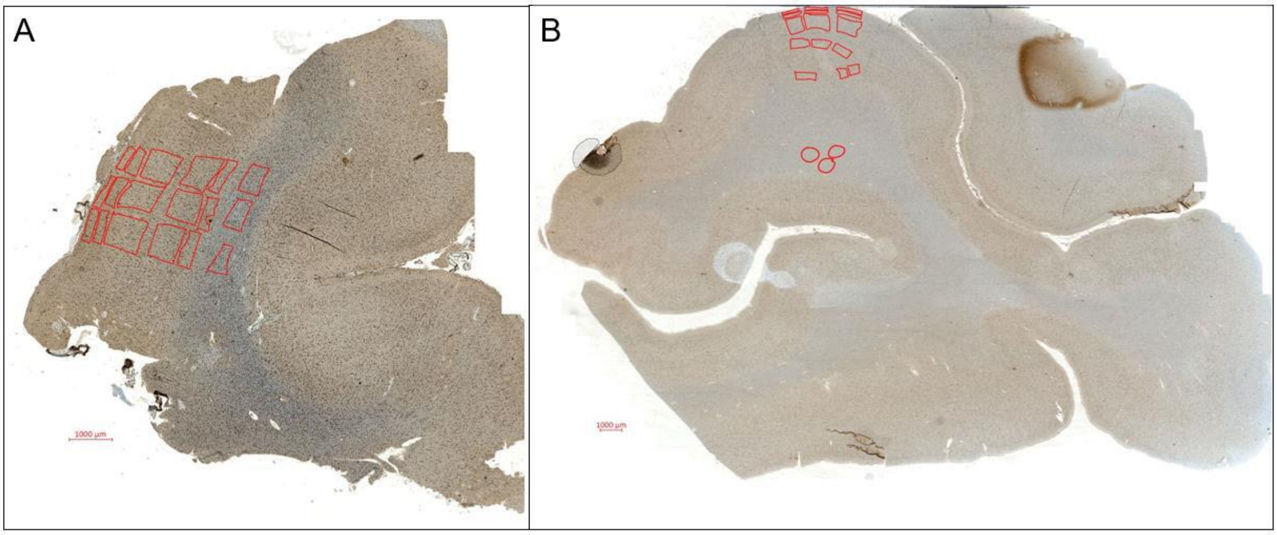

Fig SI25. Examples of light field microscopy images of temporal lobe neocortical IHC stained sections for ZBTB20. A section from a mTLE patient. **B** section from a non-epilepsy subject. The red markings are measurements in resp. Layers 1,2,3,5,6, and white matter**.**

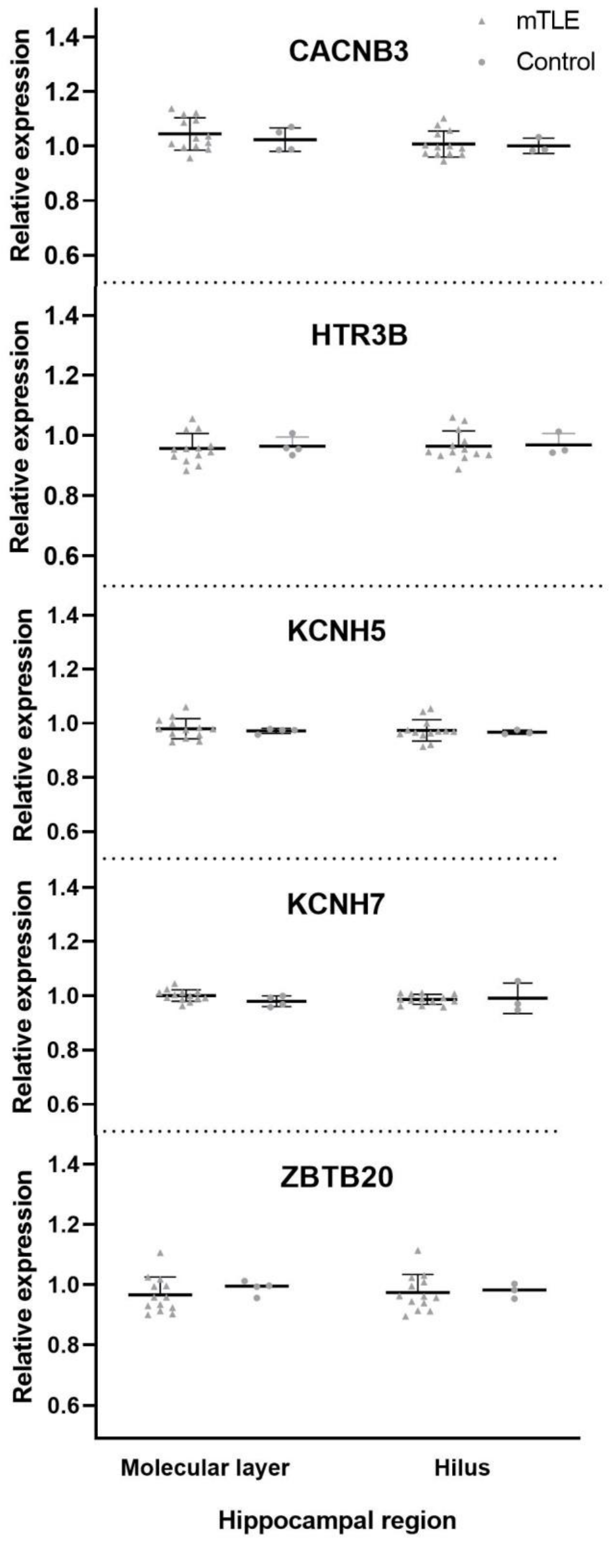

CACNB3

Region	Discovery	Adj. p-value
Molecular layer	No	0.801
Hilus	No	0.901

HTR3B

Region	Discovery	Adj. p-value
Molecular layer	No	0.926
Hilus	No	0.926

KCNH5

Region	Discovery	Adj. p-value
Molecular layer	No	0.604
Hilus	No	0.604

KCNH7

Region	Discovery	Adj. p-value
Molecular layer	No	0.248
Hilus	No	0.958

ZBTB20

Region	Discovery	Adj. p-value
Molecular layer	No	0.575
Hilus	No	0.828

Fig SI26. The relative protein level as determined by immunohistochemical analysis of CACNB3, KCNH5, KCNH7, HTR3B, and ZBTB20 in the molecular layer and hilus of the

hippocampus of mTLE patients vs. control individuals show no differences in expression Tissue from 17 mTLE patients and 16 non-epilepsy subjects were analyzed by immunohistochemistry and values represent relative expression levels as detailed in the methods section. Multiple unpaired t tests with Welch's correction and a false discovery rate of 5% were employed to test for differences between the two hippocampal regions. The mean and SD are indicated for all conditions and an adjusted p-value (adjusted for multiple testing) of <0.05 is considered significant.

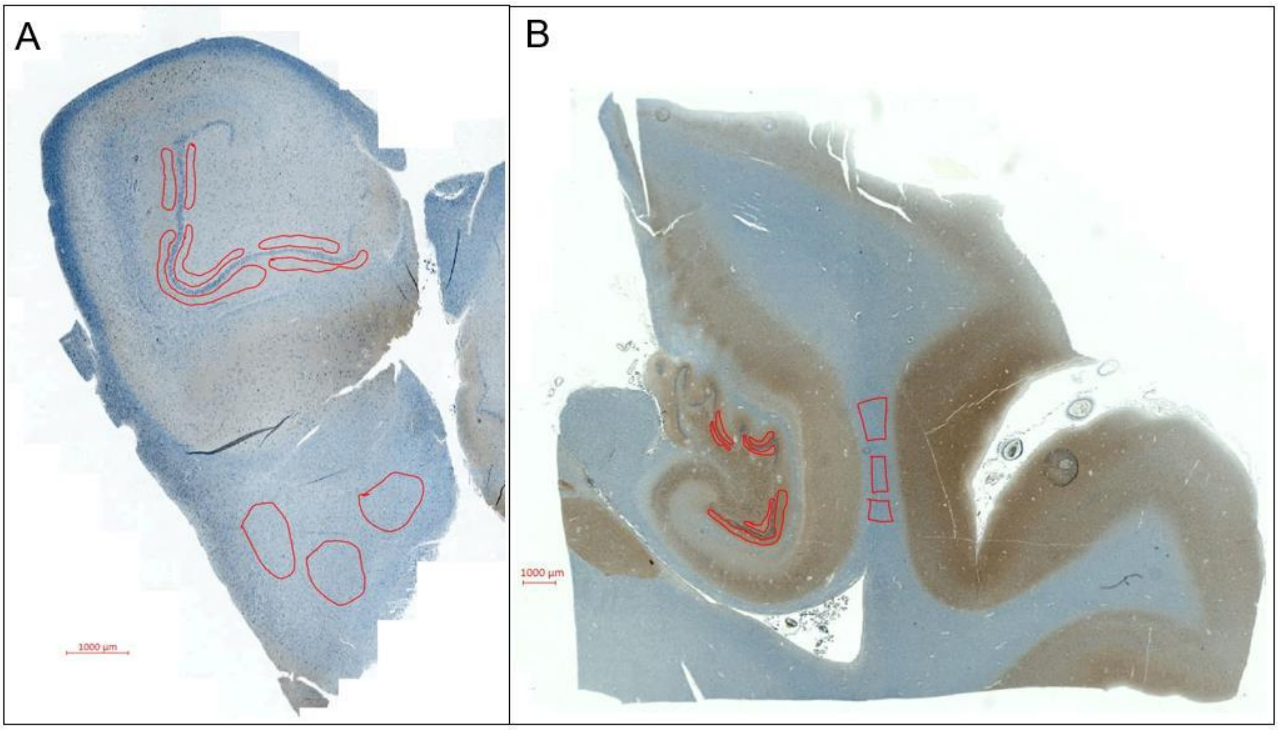

Fig SI27. Examples of light field microscopy images of hippocampal IHC stained sections for CACNB3. A section from a mTLE patient. **B** section from a non-epilepsy subject. The red markings are measurements in hilus, molecular layer and white matter**.**

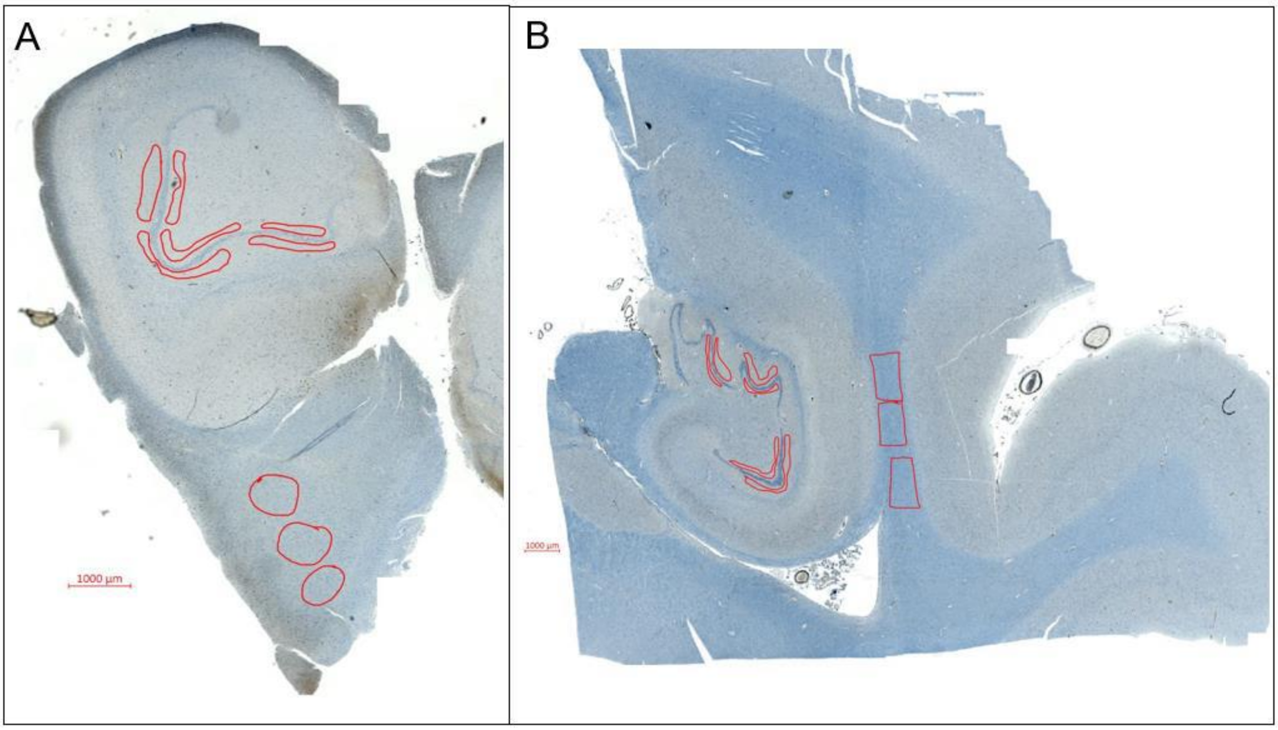

Fig SI28. Examples of light field microscopy images of hippocampal IHC stained sections for KCNH5. A section from a mTLE patient. **B** section from a non-epilepsy subject. The red markings are measurements in hilus, molecular layer and white matter**.**

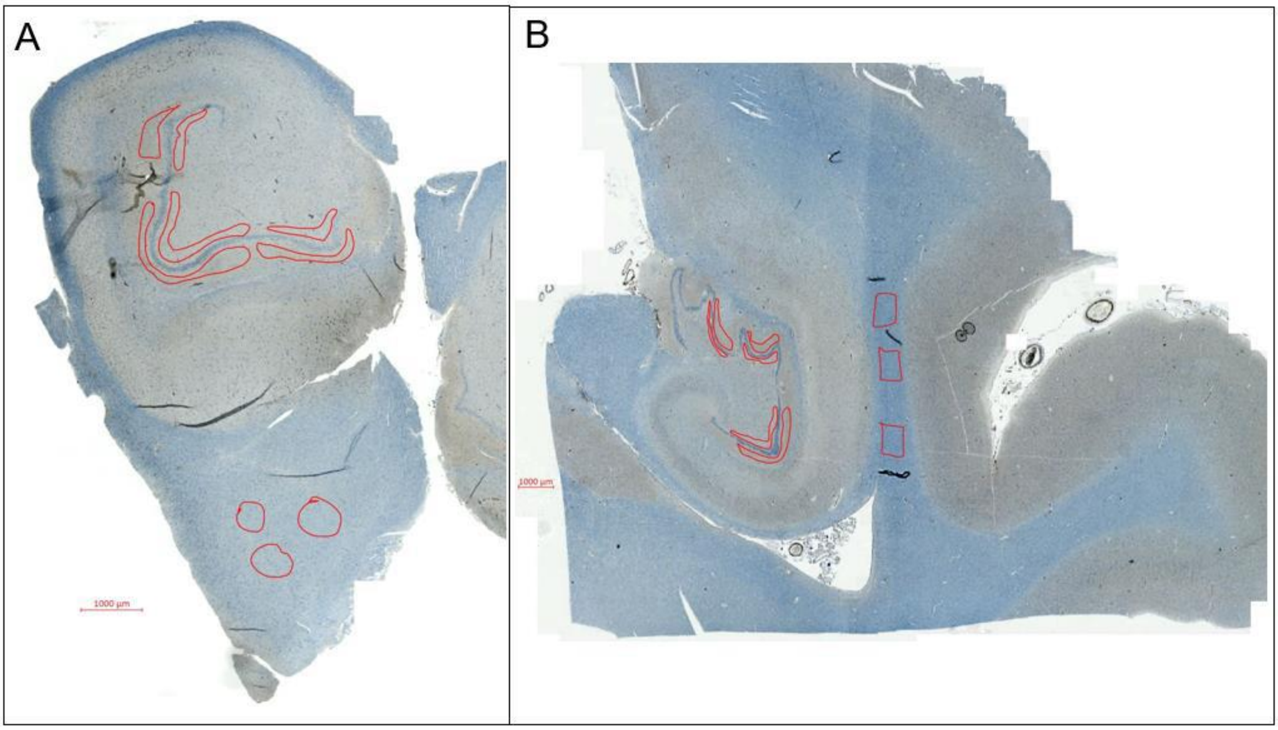

Fig SI29. Examples of light field microscopy images of hippocampal IHC stained sections for KCNH7. A section from a mTLE patient. **B** section from a non-epilepsy subject. The red markings are measurements in hilus, molecular layer and white matter**.**

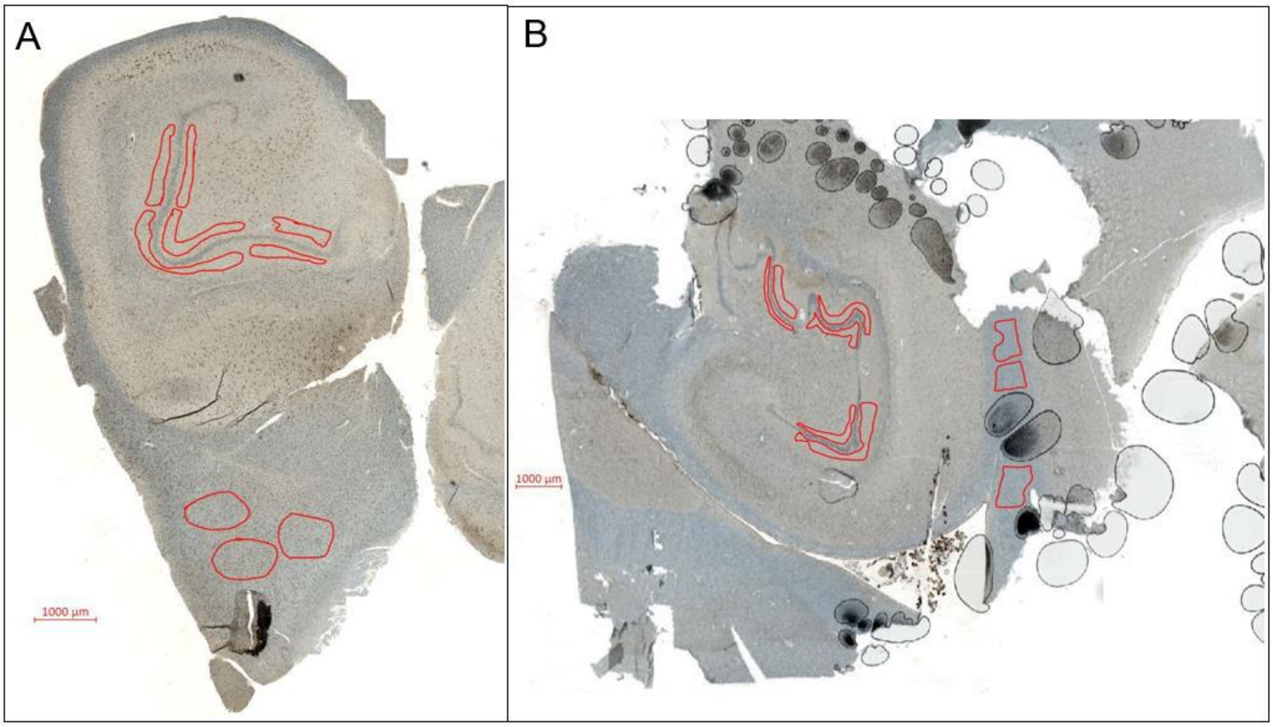

Fig SI30. Examples of light field microscopy images of hippocampal IHC stained sections for HTR3B. A section from a mTLE patient. **B** section from a non-epilepsy subject. The red markings are measurements in hilus, molecular layer and white matter**.**

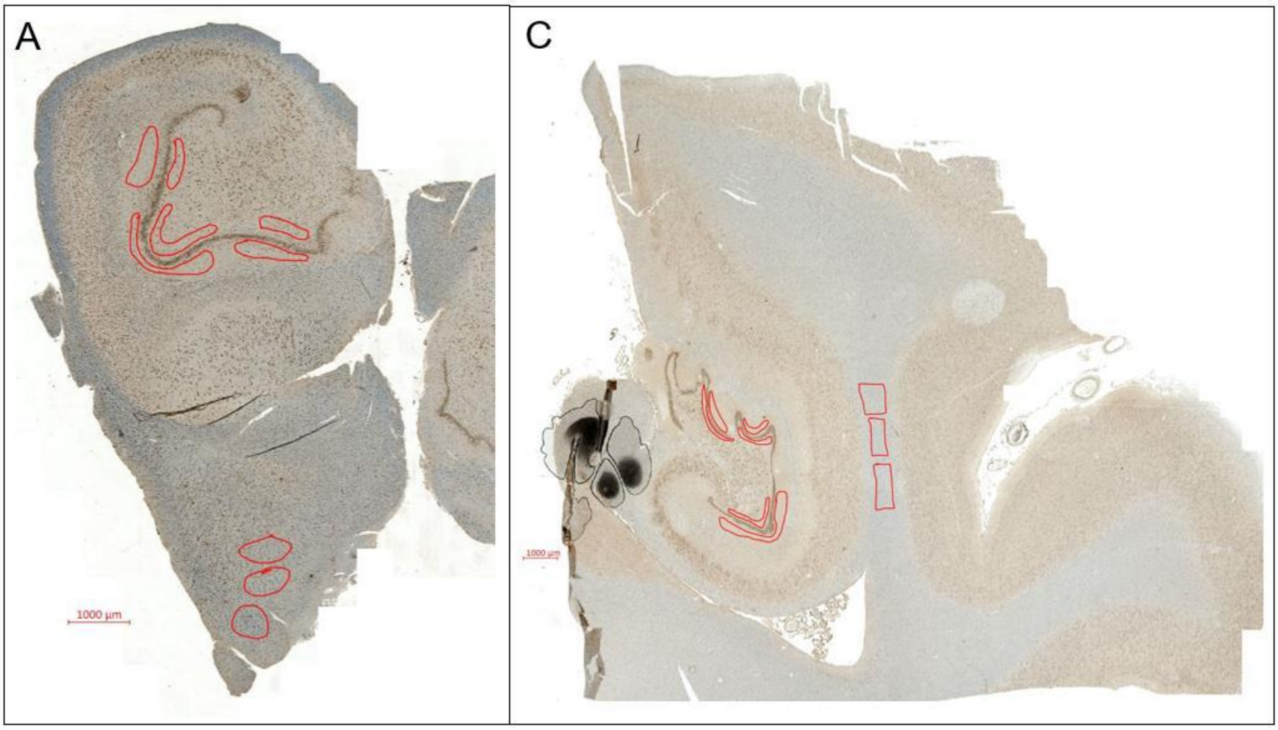

Fig SI31. Examples of light field microscopy images of hippocampal IHC stained sections for ZBTB20. A section from a mTLE patient. **B** section from a non-epilepsy subject. The red markings are measurements in hilus, molecular layer and white matter**.**

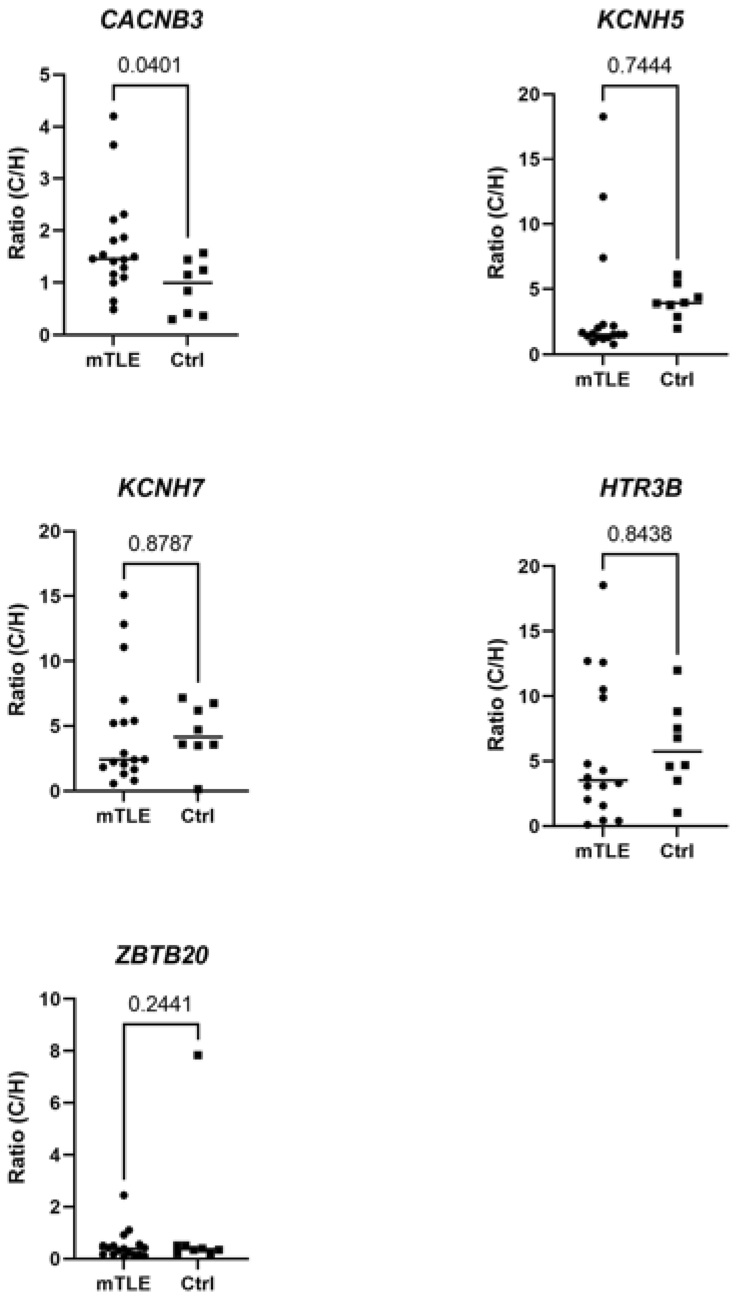

Fig SI32. mRNA expression level ratios. The ratios of the mRNA expression level between the neocortex (C) and the hippocampus (H) were calculated for the mTLE and the non-epilepsy (control; ctrl) sample groups. The calculations were based on the qPCR data presented in the main manuscript, Fig. 2. An unpaired, Student's t-test was performed, and the resulting p-values are presented. Only *CACNB3* mRNA expression ratios were significantly different between the mTLE and non-epilepsy samples.

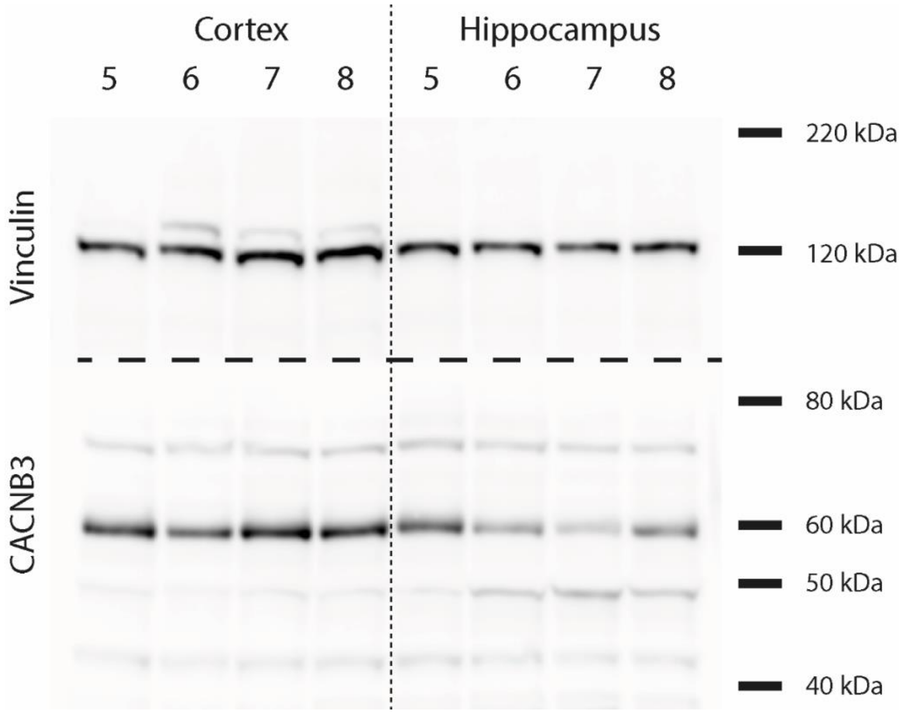

Fig SI33 Full blot showing all detected bands employed to construct panel C (mTLE) in Fig. 4 in the manuscript. See the methods and results sections in the manuscript for experimental details.

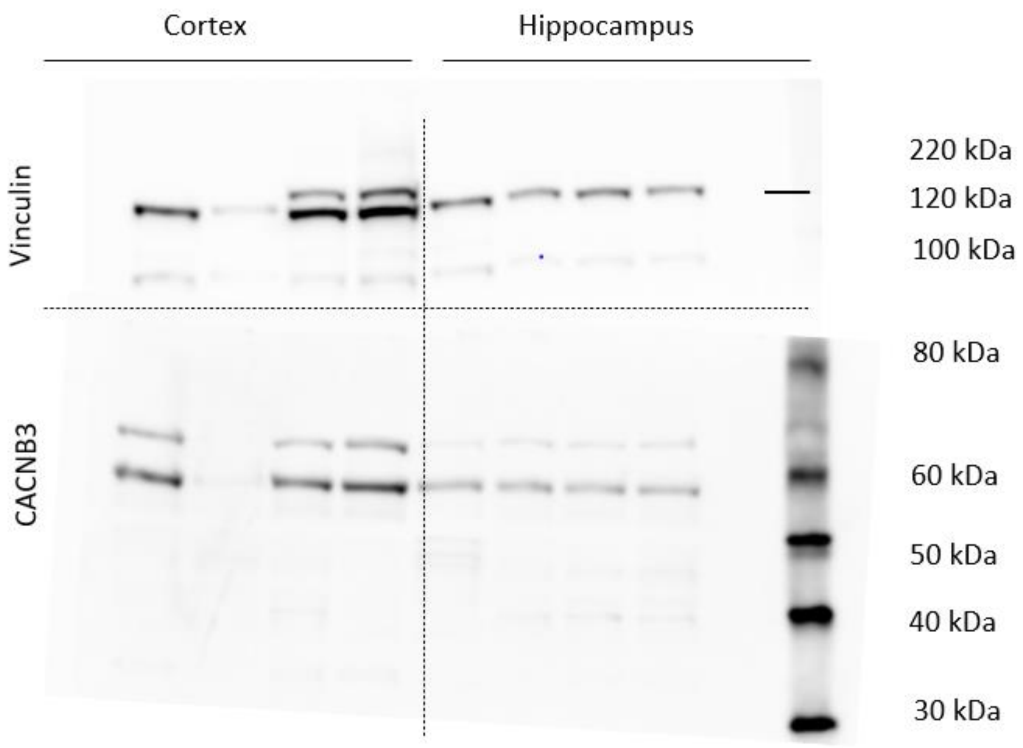

Fig SI34 Full blot showing all detected bands employed to construct panel D (non-epilepsy control) in Fig. 4 in the manuscript. See the methods and results sections in the manuscript for experimental details.

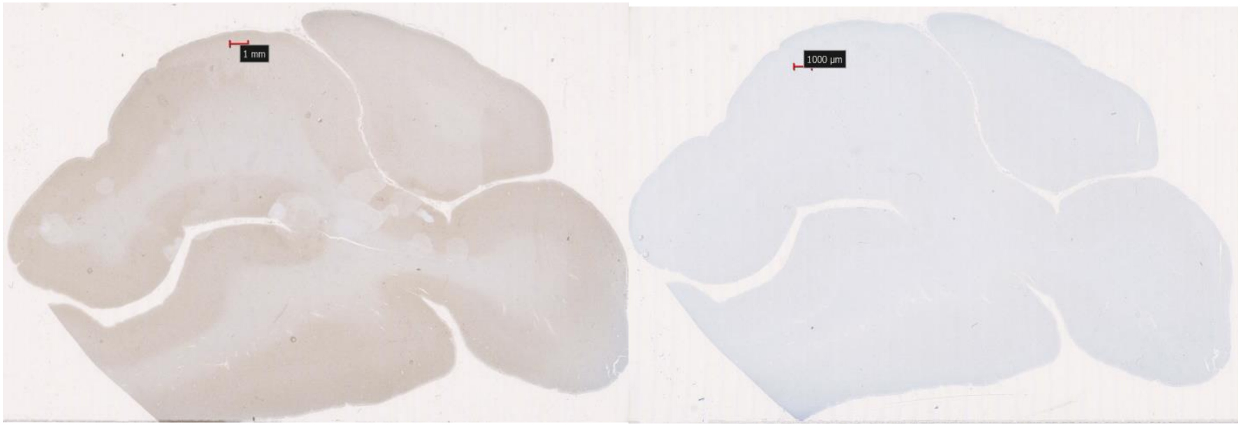

Fig SI35 Light field microscopy images (20x magnification) temporal lobe neocortical slides from a non-epilepsy subject IHC DBA+ stained for CACNB3 on the left panel and without CACNB3 on the right panel. See the methods and results sections in the manuscript for experimental details.

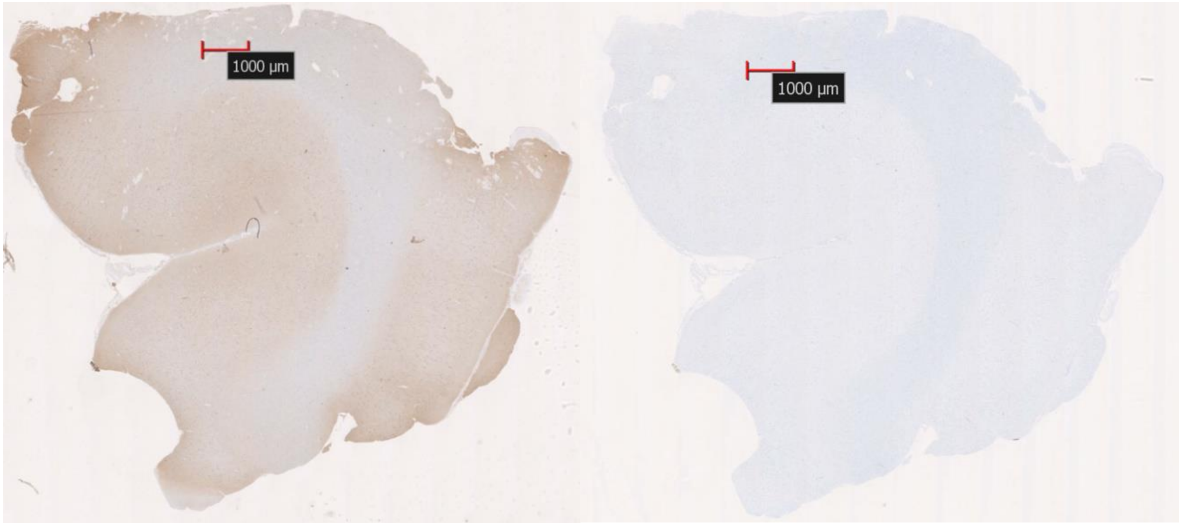

Fig SI36 Light field microscopy images (20x magnification) of mTLE temporal lobe neocortical IHC DBA+ stained slides for CACNB3 on the left panel and without CACNB3 on the right panel. See the methods and results sections in the manuscript for experimental details.

1. Scheffer IE, Berkovic S, Capovilla G, Connolly MB, French J, Guilhoto L, et al. ILAE classification of the epilepsies: Position paper of the ILAE Commission for Classification and Terminology. Epilepsia (Copenhagen). 2017;58(4):512-21.
2. Uhlén M, Fagerberg L, Hallström BM, Lindskog C, Oksvold P, Mardinoglu A, et al. Proteomics. Tissue-based map of the human proteome. Science. 2015;347(6220):1260419.
3. Grosset AA, Loayza-Vega K, Adam-Granger É, Birlea M, Gilks B, Nguyen B, et al. Hematoxylin and Eosin Counterstaining Protocol for Immunohistochemistry Interpretation and Diagnosis. Appl Immunohistochem Mol Morphol. 2019;27(7):558-63.
4. Torben M, Morten Møller f. Basal neuroanatomi. 3. udgave. ed. Moos T, Møller M, editors. Kbh: FADL; 2010.
5. Krarup S, Mertz C, Jakobsen E, Lindholm SEH, Pinborg LH, Bak LK. Distinct effects on cAMP signaling of carbamazepine and its structural derivatives do not correlate with their clinical efficacy in epilepsy. Eur J Pharmacol. 2020;886:173413.
